# Strategic communication to strengthen nurses’ professional identity and drive cultural change in healthcare organizations: A JBI scoping review protocol

**DOI:** 10.1371/journal.pone.0359546

**Published:** 2026-09-29

**Authors:** Carmen Arteaga-Jordá, María Jesús Yáñez-Galdames, Hildegart González-Luis, Cristina Oroviogoicoechea, María José Hernández-Leal

**Affiliations:** 1 Clínica Universidad de Navarra, Madrid, Spain; 2 Faculty of Economics and Business, International University of La Rioja (UNIR), Logroño, Spain; 3 School of Nursing, University of Navarra, Pamplona, Spain; 4 Navarra Institute for Health Research (IdiSNA), Pamplona, Spain; 5 Center for Health Policy and Media Engagement, George Washington University School of Nursing, Washington, District of Columbia, United States of America; 6 Clínica Universidad de Navarra, Pamplona, Spain; University of Saskatchewan, CANADA

## Abstract

Strategic communication plays a vital role in healthcare organizations undergoing continuous cultural transformation, yet its impact on reinforcing nurses’ professional identity remains underexplored. Nurses’ professional identity—how they perceive and enact their roles shaped by professional values and social interactions—is fundamental to job satisfaction, resilience, collaboration, and retention. This scoping review protocol outlines a study designed to systematically map and synthesize existing literature on the interaction between strategic communication, professional identity, and cultural change in nursing contexts. Following Joanna Briggs Institute methodology and PRISMA-ScR guidelines, a comprehensive search will be conducted across seven bibliographic databases and multiple grey literature sources for records published from 2000 to 2025, with no language restrictions. Studies will be included if they demonstrate an explicit interaction between these three core concepts, rather than their isolated presence. Two independent reviewers will conduct screening and data extraction. Data extraction and analysis will be guided by three established frameworks: a 14-component strategic communication framework, the ISPIN-IBIN nursing professional identity model, and an eight-dimension cultural transformation framework. Data will be analyzed descriptively and thematically to create an evidence map of communication models, strategies, and actions that strengthen nurses’ professional identity and support sustainable cultural transformation. An interpretative layer will also be incorporated to explore relationships between communication practices, identity construction, and organizational change. The review aims to identify implementation challenges, contextual enablers, and gaps in current knowledge. Findings will offer evidence-informed guidance for nursing leadership, education, and policy to embed identity-driven communication strategies in organizational change initiatives. Ultimately, this work seeks to advance theoretical understanding and practical application of strategic communication to support resilient nursing workforces and improve healthcare organizational culture.

## Introduction

Healthcare organizations are complex, dynamic systems undergoing continuous transformation. Within these environments, nurses’ professional identity is central to effective practice. The International Society for Professional Identity in Nursing (ISPIN) defines professional identity as “a sense of oneself, and in relationship with others, that is influenced by characteristics, norms, and values of the nursing discipline, resulting in an individual thinking, acting, and feeling like a nurse” [1, p.201]. Conceptualized as a dynamic and evolving construct, professional identity is shaped through interpersonal interactions, leadership discourse, and institutional narratives [2,3]. It underpins outcomes such as job satisfaction, resilience, interprofessional collaboration, and workforce retention [4,5]. Conversely, organizational change processes that fail to support professional identity may contribute to dissonance, disengagement, and resistance to innovation [6,7]. Yet, the mechanisms through which professional identity is reinforced during cultural transformation remain insufficiently examined.

Cultural transformation involves both structural dimensions (e.g., leadership, policies, governance) and symbolic ones (e.g., language, values, symbols) [8]. Strategic communication functions as a primary symbolic driver by aligning messaging with institutional goals and stakeholder interests to create shared meaning, strengthen symbolic coherence, and embed new practices [9–12]. While leadership models, organizational structures, and outcome metrics in healthcare change have been extensively studied [13–16], there is a lack of practical guidance on how strategic communication is operationalized to support professional narratives, stakeholder engagement, and cultural alignment.

Despite theoretical acknowledgment of communication’s role in organizational change, healthcare research has paid limited attention to strategic communication as an enabler of cultural transformation. The American Nurses Credentialing Center’s Magnet Recognition Program exemplifies this gap. Evidence shows that Magnet-designated hospitals strengthen nursing professional identity through structural empowerment, transformational leadership, and professional practice excellence [17–20]. These organizations demonstrate enhanced professional autonomy, nursing status, and engagement [21–23]. However, the literature focuses largely on structural and outcome indicators, with minimal exploration of the communicative processes that embed and sustain these transformations.

The intersection between strategic communication and nurses’ professional identity is therefore poorly conceptualized and empirically underdeveloped. This review positions itself at the crossroads of nursing, health communication, and organizational development—domains that remain rarely integrated. A scoping review is well suited to map available evidence, clarify conceptual frameworks, and synthesize practices that reinforce nursing identity during organizational change [24].

This study is grounded in the ISPIN-IBIN framework, which emphasizes embracing professional identity, communicating a unified brand image, and fostering brand awareness to promote accurate, consistent, and empowering representations of nursing—both within healthcare organizations and in the broader public sphere [25]. Building on this perspective, the review examines how strategic communication contributes to identity reinforcement during cultural transformation. While the framework highlights the importance of unified brand imagery, empirical evidence documenting its implementation in cultural transformation processes remains limited.

Accordingly, this review will include empirical and conceptual studies (qualitative, quantitative, mixed-methods), theoretical literature and grey sources. Based on the PCC (Participants–Concept–Context) framework, inclusion criteria are defined as follows:

*Participants:* Nurses in clinical, managerial, educational, leadership roles, and professionals (e.g., communication specialists, consultants, leaders) working with or on behalf of nurses to support their professional identity during cultural transformation.
*Concept:* Professional identity shaped through strategic communication, understood as institution-aligned process that reinforces professional identity and drives cultural transformation.
*Context:* Healthcare organizations undergoing professional or cultural transformation.

In summary, healthcare organizations require a clearer understanding of how strategic communication strengthens nurses’ professional identity during cultural transformation. Without such insights, change initiatives risk misalignment and weakened professional cohesion. This scoping review will systematically chart the extent, range, and nature of evidence on strategic communication in nursing cultural change, with attention to the ISPIN-IBIN Framework’s domain “Communicate a Unified Brand Image”. Findings will contribute to theoretical development and provide practical guidance for embedding communication strategies that reinforce nursing identity and enable sustainable organizational transformation.

### Review question

This scoping review seeks to answer the following primary question:

*How strategic communication aimed at strengthening nurses’ professional identity contributes to driving cultural transformation within healthcare organizations?*

In line with the PCC framework, the review will address the following sub-questions:

1. What models, strategies, plans and actions of communication have been implemented in healthcare organizations to support cultural change while reinforcing nurses’ professional identity?
2. How does the literature describe the relationship between strategic communication and the development or reinforcement of nurses’ professional identity in the context of cultural transformation?
3. What conceptual or empirical gaps exist that may hinder the design or implementation of strategic communication strategies for cultural transformation in nursing?

### Inclusion criteria

#### Participants.

Studies must focus on nurses as primary participants or key stakeholders in organizational cultural change within healthcare settings. Eligible participants include nursing teams, frontline clinical nurses, nurse leaders, administrative nurses, educators, and representatives of professional nursing associations. Interdisciplinary team studies are eligible only if nurse-specific data are clearly reported or can be disaggregated for analysis of nursing identity, professional development, or communication practices.

Additionally, studies involving communication professionals, consultants, leaders, or other stakeholders whose actions explicitly support or shape nurses’ professional identity during cultural transformation processes will be included.

#### Concepts.

This review examines three interrelated concepts—strategic communication, professional identity, and cultural transformation—and applies operational frameworks to support a structured and coherent analysis.

Studies will be eligible if they demonstrate an explicit interaction between the three core concepts, rather than their isolated presence. Specifically, included studies must reflect a relationship in which strategic communication operates as an intentional organizational mechanism within processes of institutional cultural transformation, influencing the construction or reinforcement of nurses’ professional identity.

#### Operationalization of strategic communication.

Strategic communication is defined as a purposeful, institution-aligned process that enables organizational alignment and transformation while reinforcing nurses’ professional identity—that is, how nurses perceive, articulate, and enact their roles and values within institutional settings. For the purpose of this review, it is operationally defined as intentional, organization-level communication practices aligned with institutional goals and designed to influence professional identity and organizational culture. In the absence of an established operational model, a 14-component framework was developed via integrative synthesis of scholarship on organizational culture, change management, and communication [9,10,26–32], and refined through expert consultation and pilot testing. Components range from contextual diagnosis and identity alignment to participatory spaces, leadership involvement, and institutionalization of communication practices (see S1 Appendix). These components primarily guide data extraction and analysis, supporting a structured identification and categorisation of communication practices.

#### Anchoring in the ISPIN-IBIN Framework.

To ensure both theoretical rigor and practical relevance, the review is explicitly structured around the ISPIN-IBIN Framework’s attribute “Communicate a Unified Brand Image”, which promotes accurate, consistent, and empowering representations of nursing [4,25] (see S2 Appendix). It is further guided by ISPIN’s four-domain model of nursing professional identity—Values and Ethics; Knowledge and Skills; Nurse as Leader; and Professional Comportment [4,33]. This dual anchoring provides a comprehensive lens for examining the interplay between professional identity and organizational branding within healthcare transformation.

#### Operationalization of cultural transformation.

Cultural transformation is understood as an intentional, sustained, multidimensional process involving structural elements (leadership, governance, policies, practices) and symbolic elements (values, narratives, professional identity) [8,29,34]. An eight-dimension operational framework—enculturation and professional socialization [2,23], transformational leadership [12,22], participatory governance [24–26], organizational learning [27–29], culture of safety [30,31], diversity and inclusion [32,33], implementation and integration [29,34,35], and sustained outcomes [36–38]—was derived from integrative synthesis of organizational change and healthcare culture scholarship (see S3 Appendix). This framework distinguishes cultural transformation from purely technical or procedural change and guides data extraction.

In summary, by applying three complementary frameworks—strategic communication (how practices and messages are enacted), professional identity (how nursing identity is shaped), and cultural transformation (how organizational change is structured and sustained)— this review establishes a robust conceptual foundation for study selection and a structured approach to data extraction and analysis. The frameworks will be used complementarily but with distinct functions: the strategic communication model will support the identification and categorisation of communication practices; the ISPIN-IBIN framework will inform the interpretation of professional identity dimensions; and the cultural transformation framework will provide the organizational context for analysis.

### Context

The review will include studies conducted in healthcare organizations —hospitals, clinics, primary care centers, and other institutional settings— undergoing or planning cultural organizational change. Both public and private contexts will be considered.

For clarity, the eligibility criteria derived from these conceptual foundations are summarised in Table 1 following the PCC framework.

**Table 1:** PCC inclusion and exclusion summary.

Domain	Inclusion	Exclusion
Participants	Studies involving nurses engaged in organizational or cultural change processes (e.g., frontline nurses, nurse leaders, educators, or nursing association representatives). Interventions or communication strategies may be implemented by nurses, communication professionals, organizational leaders, consultants, or other stakeholders, provided that the target population or intended impact explicitly relates to nurses’ professional identity, professional development, or communication practices. Interdisciplinary studies are eligible only if nurse-specific data are clearly reported or can be disaggregated for analysis.	Studies involving multidisciplinary healthcare professionals where nurse-specific data cannot be identified or disaggregated for analysis of nursing identity, professional development, or communication practices, as well as studies focused exclusively on nursing students.
Concept	Studies examining strategic communication as an intentional organizational mechanism within processes of cultural transformation, where communication practices are explicitly linked to the development, strengthening, or enactment of nurses’ professional identity. Eligible studies must demonstrate an interaction between strategic communication, cultural transformation, and professional identity, rather than addressing these concepts in isolation. Flexible judgment will allow inclusion where terminology varies but the conceptual relationship is evident.	Communications that are purely technical or generic, lack strategic intent, do not address professional identity, or are not situated within processes of cultural transformation; and studies where the three core concepts are not meaningfully and explicitly connected.
Context	Healthcare organizations undergoing or planning cultural organizational change (e.g., hospitals, clinics, primary care centers, long-term care), in both public and private sectors.	Non-healthcare or unrelated organizational settings.

*Note.* PCC = Participants–Concept–Context*. Source:* own elaboration

### Types of sources

This scoping review will include empirical, conceptual, and opinion literature relevant to strategic communication, professional identity, and cultural transformation in healthcare. Eligible sources are:

- Qualitative studies (e.g., phenomenology, grounded theory, ethnography, case study, action research, qualitative description).
- *Quantitative studies* (e.g., cross-sectional, correlational, cohort, case-control).
- Mixed-methods studies addressing communication practices, professional identity, or cultural change.
- *Conceptual and theoretical papers* (frameworks, models, institutional reports, and grey literature).
- *Evidence syntheses* (scoping reviews, systematic reviews, meta-syntheses) that meet inclusion criteria and advance conceptual understanding.
- *Editorials, commentaries, and opinion pieces* when they provide conceptual clarity or practical insights; these will not be treated as empirical evidence and will be analysed separately from empirical studies and clearly identified in the synthesis.

## Methods

This review will follow the Joanna Briggs Institute (JBI) methodology for scoping reviews [35,36] and report according to PRISMA-ScR [37]. The protocol is registered on the Open Science Framework, with any amendments documented publicly [38]. Relevant PRISMA-P items [39] have been adapted to improve transparency (see S4 Appendix), in accordance with current guidance for reporting scoping review protocols.

The methodological process underpinning the design and planning of this scoping review is illustrated in Fig 1. This flow diagram summarizes the sequential phases, key decision points, and validation steps undertaken to ensure methodological rigor and transparency. A tentative timeline for the different phases of the scoping review is provided in Supplementary Information (S5 Appendix).

**Fig 1:**
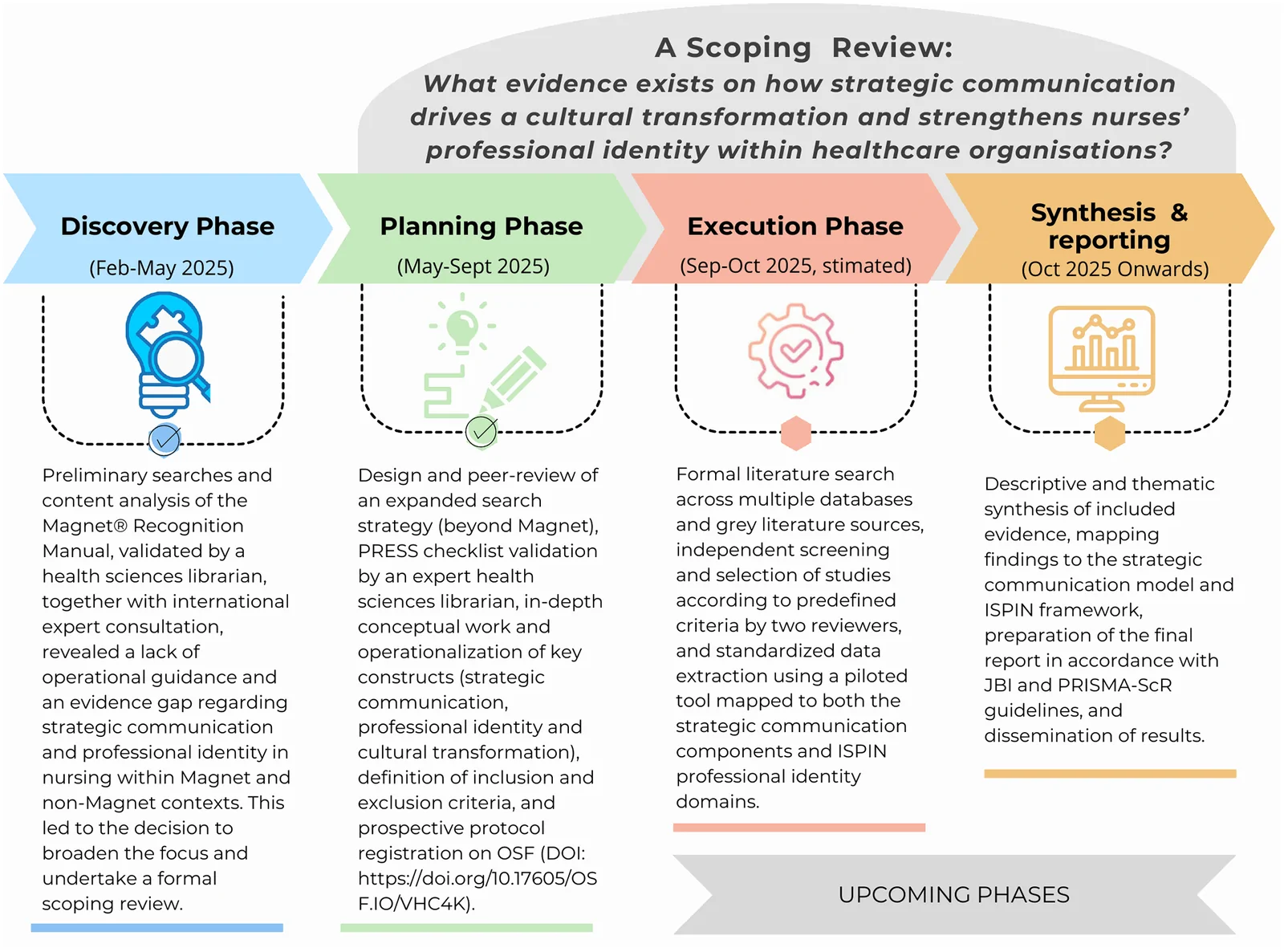
Flow diagram of the scoping review process. Sequential phases, key decision points, and validation steps.

### Search strategy

An initial limited search of MEDLINE (via PubMed), CINAHL (via EBSCO), and Web of Science informed the full search strategy, developed with a university librarian and peer-reviewed via PRESS [40]. The final search strategy combines terms related to nursing identity, organizational transformation, and strategic communication, limited to studies published between 2000 and 2025; a period reflecting the development of structured strategic communication practices and identity frameworks in healthcare. Boolean operators and MeSH terms were used to maximise sensitivity and capture both controlled vocabulary and free-text variations (S6 Appendix)

The main search will be conducted in seven bibliographic databases and be complemented by grey literature searches (see Table 2). To enhance transparency and reproducibility, database-specific adaptations of the search strategy are provided in S7 Appendix. Reference lists of included studies and relevant reviews will be screened (snowballing). No language restrictions will apply; non-English/Spanish records will be machine-translated for screening and full-text review. A preliminary search of Cochrane, JBI Evidence Synthesis, PROSPERO, and OSF found no existing or ongoing scoping reviews on this topic.

**Table 2:** Information sources by type.

Source Type	Databases / Platforms
Bibliographic	MEDLINE (PubMed), CINAHL (EBSCO), Scopus, Web of Science Core Collection, APA PsycINFO (EBSCO), Dialnet, SciELO.
Grey literature	OpenGrey, ProQuest Dissertations & Theses Global-

*Source:* Own elaboration.

### Study/Source of evidence selection

Records will be managed in Covidence for deduplication and blinded screening. Two reviewers will independently screen titles/abstracts and full texts against eligibility criteria, with disagreements resolved by consensus or a third reviewer. Exclusion reasons at full-text stage will be documented. References for included studies will be managed in Mendeley (Elsevier, 2025). The process will be illustrated with a PRISMA-ScR flow diagram [37].

#### Data extraction.

Two reviewers will independently extract data using a standardized tool aligned with the three operational frameworks: the 14-component strategic communication model, the four-domain ISPIN-IBIN professional identity framework, and the eight-dimension cultural transformation framework (see S8 Appendix). The instrument includes extraction fields, operational definitions, coding instructions, and an illustrative example of application. Calibration on 25 studies yielded a Cohen’s κ = 0.83, exceeding the κ ≥ 0.75 threshold. Authors will be contacted for missing or unclear information. No formal quality appraisal will be conducted, per JBI guidance.

### Data analysis and presentation

Extracted data will undergo descriptive (quantitative) and thematic (qualitative) analysis. Quantitative data (e.g., publication year, country, study design, setting) will be summarized in tables and figures to map the evidence. Qualitative data will be coded reflexively to identify themes related to strategic communication, professional identity, and cultural transformation [41]. A glossary of key terms will be developed, and inclusion criteria applied flexibly to accommodate diverse conceptualizations.

Findings will be integrated via a convergent synthesis approach, mapping quantitative, qualitative, and theoretical evidence onto the three frameworks. Methodological heterogeneity and its implications will be reported. Conceptual models or evidence-mapping diagrams may be developed to illustrate relationships among communication strategies, contextual factors, and outcomes. Knowledge gaps and underexplored areas will be explicitly identified. Beyond descriptive mapping, the review will incorporate an interpretative layer to explore relationships between communication practices, identity construction, and cultural transformation.

In this context, the multidisciplinary composition of the research team—encompassing expertise in strategic communication, clinical leadership in cultural transformation processes associated with Magnet accreditation, and doctoral-level research training—is considered a key strength. To further enhance analytical rigor, an external expert consultation will be conducted following the preliminary synthesis and prior to the final interpretation of findings. One independent international expert with recognized expertise in nursing leadership, professional identity, strategic communication, or organizational transformation will be invited to review the preliminary synthesis. Expert selection will be based on demonstrated expertise through scholarly publications, leadership experience, and contributions to research or practice in one or more of these fields. Feedback will be collected through written comments and/or a virtual consultation, focusing on conceptual coherence, interpretation of findings, and practical applicability. Relevant feedback will be considered during refinement of the discussion and conceptual integration of findings; however, it will not modify the study eligibility criteria, extracted data or mapped evidence.

### Expected outcomes

This scoping review will produce a comprehensive evidence map of how strategic communication contributes to cultural transformation and the strengthening of nurses’ professional identity in healthcare organizations. Results will be presented in descriptive tables and narrative summaries, systematically mapped to the 14-component strategic communication model, the four-domain ISPIN-IBIN framework, and the eight-dimension cultural transformation framework. Where appropriate, thematic analysis will illuminate patterns, mechanisms, and research gaps.

The review is expected to:

- Identify and categorise models, strategies, plan and actions of communication used in healthcare settings to develop, reinforce, or shape nurses’ professional identity during cultural transformation.
- Reveal opportunities for health organizations and educators to leverage the ISPIN-IBIN Framework or other models as a catalyst for increasing professional identity awareness and fostering a unified brand image in nursing within the context of cultural transformation.
- Offer recommendations for future research, policy, and organizational practice in nursing leadership and workforce development

### Implications for practice, policy and research

The findings of this scoping review will provide evidence-informed guidance for nursing leaders, educators, and healthcare managers implementing cultural organizational change. By systematically mapping how strategic communication intersects with cultural transformation and professional identity, the review will equip stakeholders with actionable strategies while highlighting implementation challenges commonly encountered in practice.

Key implications include:

- Practice: Supporting the design and implementation of communication strategies that foster professional identity cohesion, resilience, and excellence in nursing practice.
- Policy: Providing tangible recommendations—grounded in the ISPIN Framework— for embedding strategic communication into leadership development curricula, educational programs, and health policy frameworks that strengthen nursing identity.
- Research: Opening new avenues for empirical investigation at the intersection of strategic communication, professional identity, and organizational change in healthcare contexts.

This review will contribute to theory-building by mapping current evidence, delineating core components and conceptual linkages, and identifying research gaps. The findings will establish a foundation for future empirical studies focused on identity-driven communication strategies in nursing and healthcare leadership

## Strengths and limitations

This section outlines the key methodological advantages and potential constraints of the proposed scoping review, highlighting factors that support the rigor and relevance of the findings as well as considerations that may influence their interpretation [24,36,37,42].

*Strengths:*

- Adherence to established methodological standards (JBI framework, PRISMA-ScR guidelines).
- Application of a robust tri-dimensional analytical framework enabling multidimensional, practice-oriented synthesis.
- Comprehensive search strategy across seven databases, grey literature inclusion, and absence of language restrictions, enhancing comprehensiveness and reducing publication bias.
- Methodological rigour is further ensured through piloting, dual independent screening/extraction, and iterative refinement of tools and criteria.
- Integration of the ISPIN-IBIN Framework adds analytical rigor and practical relevance, revealing concrete opportunities for embedding identity-focused communication in leadership, education, and policy initiatives.

*Limitations:*

- As a scoping review, no formal quality appraisal will be conducted, potentially limiting interpretability of findings.
- Conceptual heterogeneity across studies may reduce comparability and pose synthesis challenges.
- Inconsistent terminology or indexing may lead to the inadvertent exclusion of relevant literature, despite the comprehensive search strategies.
- The review will map and categorise communication strategies without assessing effectiveness or impact, though studies evaluating effectiveness will be included where relevant.
- Given the conceptual novelty and interdisciplinary complexity, the volume of eligible studies may be limited, potentially constraining breadth of available and transferability across diverse healthcare settings.

Potential sources of bias inherent to scoping reviews should be acknowledged. Consistent with JBI methodology, this review will not undertake a formal critical appraisal of included sources, as its primary purpose is to map the breadth and characteristics of the available literature. Nevertheless, the methodological characteristics and nature of the included sources will be considered when interpreting the mapped evidence. Publication bias, language bias, and conceptual heterogeneity may also influence the resulting evidence map despite the inclusion of grey literature and the absence of language restrictions. These potential sources of bias will be mitigated through a comprehensive peer-reviewed search strategy, independent dual screening and data extraction, calibration procedures, and predefined coding frameworks to enhance transparency, consistency, and reproducibility.

Despite these methodological limitations and potential sources of bias, the rigorous methodological approach adopted—including adherence to JBI guidance, independent dual review processes, and transparent reporting—is expected to provide a credible and comprehensive synthesis of the available evidence. The resulting evidence map is expected to provide meaningful guidance for nursing leadership and policy development, stimulate further research, and support systematic integration of strategic communication and professional identity within nursing-led organizational transformation.

## Ethics and dissemination

This scoping review requires no ethical approval as it involves only publicly available documents and published literature, with no primary or personal data collection.

Results will be disseminated through:

- Publication in a peer-reviewed journal.
- Presentations at relevant academic and professional conferences.
- Stakeholder briefings for nursing leadership, organizational development, and health communication communities.

The protocol has been prospectively registered on the Open Science Framework (OSF): https://doi.org/10.17605/OSF.IO/VHC4K

## Supporting information

S1 Appendix Strategic communication framework. Conceptual model comprising 14 components to guide the analysis of strategic communication practices.
(PDF)

S2 Appendix Domains of professional identity. Detailed description of ISPIN-IBIN’s four domains of nursing professional identity.
(PDF)

S3 Appendix Cultural transformation framework. Operational framework outlining eight key dimensions of cultural change in healthcare organizations.
(PDF)

S4 Appendix PRISMA-P 2015 checklist modified for the scoping review protocol. (PDF)

S5 Appendix Tentative scoping review timeline. (PDF)

S6 Appendix Full search strategy. Complete database search strings, PRESS peer-review documentation, and OSF DOI for protocol registration.
(PDF)

S7 Appendix Database search adaptation framework. (PDF)

S8 Appendix Data extraction instrument and coding guide. Standardized and piloted data extraction instrument adapted from JBI methodology, including extraction fields, operational definitions, coding guidance, and an illustrative example of application.
(PDF)

S9 Appendix References for supporting information. (PDF)
