# Supplementary material for: Strategic communication to strengthen nurses’ professional identity and drive cultural change in healthcare organizations: A JBI scoping review protocol
Source: PLoS One. 2026 Sep 29;21(9):e0359546. doi: 10.1371/journal.pone.0359546 (PMC13623099; doi:10.1371/journal.pone.0359546)
Supplement: Supplementary file 1 [file pone.0359546.s001.pdf]

S1 Appendix. Strategic Communication Framework

Strategic Communication Framework

Nº	Component	Concise definition
1	Organizational-context diagnosis	Systematic assessment of the real cultural climate (values, symbols, readiness) before designing messages [1,2].
2	Mission-, vision- & identity alignment	Explicit linkage of all communication to the organization's purpose and desired professional identity [3–6].
3	Structured, transversal communication plan	Written, time-bound plan that integrates goals, audiences, channels, responsibilities and metrics across departments [3,6,7].
4	Cultural adaptation & audience segmentation	Tailoring of tone, content and format to diverse internal publics (units, shifts, professions) for higher salience [1,2,8].
5	Spaces for participation & active listening	Regular two-way forums (town-halls, shared-governance councils, digital feedback loops) that empower staff voices [9,10.].
6	Coherent, shared narrative	A unifying story that connects past strengths to future aspirations, repeated consistently by leaders and staff [3,4]
7	Recognition & visibility of nursing	Symbolic and media actions that highlight nurses' contributions, reinforcing pride and identity [5,8,9,11].
8	Involvement of institutional leadership	Continuous, credible presence of executives (CNO, CEO) as chief communicators and role-models [3,6].
9	Narrative consistency across channels	Alignment of wording, imagery and data in emails, social media, intranet, meetings, signage, etc [3,14.].

Nº	Component	Concise definition
10	Mechanisms to evaluate communication impact	Use of surveys, analytics and outcome dashboards to measure reach, understanding and behaviour change [[3,6].
11	Flexibility & continuous improvement	Agile review cycles that adjust messages and tactics in response to feedback and evolving context [6,7].
12	Ethical adaptation & diversity sensitivity	Ensuring messages respect cultural, gender and linguistic diversity and adhere to professional ethics [2,5,8,9,15].
13	Institutionalization of communication strategies	Embedding roles, policies and budgets so practices persist beyond individual champions [3,7].
14	Explicit intent for sustainable cultural change	Framing communication as a long-term driver of deeper norms—not a one-off campaign [16].

Source. Own elaboration based on a synthesis of relevant literature and analytical judgment. Author names refer to foundational sources used in the development of each component. Full citations are provided in the reference list.

Note: References for this section are provided in S8 Appendix: References for Supporting Information.
