# Supplementary material for: Strategic communication to strengthen nurses’ professional identity and drive cultural change in healthcare organizations: A JBI scoping review protocol
Source: PLoS One. 2026 Sep 29;21(9):e0359546. doi: 10.1371/journal.pone.0359546 (PMC13623099; doi:10.1371/journal.pone.0359546)
Supplement: Supplementary file 2 [file pone.0359546.s002.pdf]

S2 Appendix. Domains of professional identity

Domains of professional identity

Nº	ISPIN Domain	Brief Description
1	Values and ethics	Core ethical principles and professional values
2	Knowledge and skills	Use and application of nursing knowledge
3	Nurse as leader	Leadership, autonomy, accountability in nursing role
4	Professional comportment	Behaviours and attitudes reflecting professional identity

Note. Domains based on Joseph & Godfrey (2023) [5]. References for this section are provided in S8 Appendix: References for Supporting Information.
