# Supplementary material for: Strategic communication to strengthen nurses’ professional identity and drive cultural change in healthcare organizations: A JBI scoping review protocol
Source: PLoS One. 2026 Sep 29;21(9):e0359546. doi: 10.1371/journal.pone.0359546 (PMC13623099; doi:10.1371/journal.pone.0359546)
Supplement: Supplementary file 3 [file pone.0359546.s003.pdf]

S3 Appendix. Cultural Transformation Framework

Cultural Transformation Framework

**Dimension**	**Definition / Focus**
Enculturation and professional socialization	Processes through which professionals are oriented and integrated into the mission, vision, and values of the organization [17,18].
Transformational leadership and coalitions for change	Leaders articulating vision, mobilizing coalitions, and generating early wins to drive change [12,16].
Participatory governance and empowerment	Structures that decentralize decision-making and give nurses a substantive voice in organizational policies and management [19–21].
Organizational learning and continuous improvement	Mechanisms that enable reflection, feedback, and adaptive learning beyond isolated interventions [21–23].
Culture of safety and just culture	Shifting from a blame culture to a just culture that promotes reporting and organizational learning from errors [24,25],
Diversity, equity, inclusion (DEI) and cultural competence	Organizational capacity to address workforce and patient diversity through inclusive policies and culturally competent care [26,27]
Implementation and integration of innovations	Moving beyond pilots to embed new practices into institutional systems, SOPs, and performance indicators [23,28,29].
Measurable and sustained outcomes	Evidence of sustained improvements in clinical, professional, and patient outcomes over time [30–32].

Source. Own elaboration based on a synthesis of relevant literature and analytical judgment. Author names refer to foundational sources used in the development of each component. Full citations are provided in the reference list.

Note: References for this section are provided in S8 Appendix: References for Supporting Information.
