# Supplementary material for: Strategic communication to strengthen nurses’ professional identity and drive cultural change in healthcare organizations: A JBI scoping review protocol
Source: PLoS One. 2026 Sep 29;21(9):e0359546. doi: 10.1371/journal.pone.0359546 (PMC13623099; doi:10.1371/journal.pone.0359546)
Supplement: Supplementary file 4 [file pone.0359546.s004.pdf]

S4 Appendix. Checklist. PRISMA-P 2015 checklist

This PRISMA-P checklist has been adapted for a scoping review, following JBI methodological guidance and the PRISMA-ScR extension. Non-applicable items to scoping reviews are marked as 'N/A'; relevant equivalents from JBI or PRISMA-ScR are referenced where appropriate.

This checklist has been adapted for use with protocol submissions to Systematic Reviews from Table 3 in Shamseer et al [33]: Preferred reporting items for systematic review and meta-analysis protocols (PRISMA-P) 2015 statement. Systematic Reviews 2015 4:1

Section/topic	#	Checklist item	Information reported		Line number(s)
			Yes	No	
**ADMINISTRATIVE INFORMATION**					
**Title**					
Identification	1a	Identify the report as a protocol of a systematic review	x	☐	1-3
Update	1b	If the protocol is for an update of a previous systematic review, identify as such	x	☐	N/A. Not an update protocol
**Registration**	2	If registered, provide the name of the registry (e.g., PROSPERO) and registration number in the Abstract	x	☐	160-165 https://doi.org/10.17605/OSF.IO/VHC4K
**Authors**					

Section/topic	#	Checklist item	Information reported		Line number(s)
			Yes	No	
Contact	3a	Provide name, institutional affiliation, and e-mail address of all protocol authors; provide physical mailing address of corresponding author	x	☐	1; Title page. Full affiliations and corresponding author clearly listed.
Contributions	3b	Describe contributions of protocol authors and identify the guarantor of the review	x	☐	pp. 20-21. Author contributions reported according to CRediT taxonomy
**Amendments**	4	If the protocol represents an amendment of a previously completed or published protocol, identify as such and list changes; otherwise, state plan for documenting important protocol amendments	x	☐	N/A. No amendments from previous versions; no planned amendments documented
**Support**					
Sources	5a	Indicate sources of financial or other support for the review	x	☐	460-465
Sponsor	5b	Provide name for the review funder and/or sponsor	x	☐	N/A. No funding or sponsorship received for this review (see lines 460–465)
Role of sponsor/funder	5c	Describe roles of funder(s), sponsor(s), and/or institution(s), if any, in developing the protocol	x	☐	N/A. No funders or sponsors involved in developing the protocol (see lines 460–465)
**INTRODUCTION**					
**Rationale**	6	Describe the rationale for the review in the context of what is already known	x	☐	10-150

Section/topic	#	Checklist item	Information reported		Line number(s)
			Yes	No	
**Objectives**	7	Provide an explicit statement of the question(s) the review will address with reference to participants, interventions, comparators, and outcomes (PICO)	x	☐	150-160
**METHODS**					
**Eligibility criteria**	8	Specify the study characteristics (e.g., PICO, study design, setting, time frame) and report characteristics (e.g., years considered, language, publication status) to be used as criteria for eligibility for the review	x	☐	280-310
**Information sources**	9	Describe all intended information sources (e.g., electronic databases, contact with study authors, trial registers, or other grey literature sources) with planned dates of coverage	x	☐	320-340
**Search strategy**	10	Present draft of search strategy to be used for at least one electronic database, including planned limits, such that it could be repeated	x	☐	Supplementary Information. S3 Appendix: Full search strategy (PRESS validated).
STUDY RECORDS					
Data management	11 a	Describe the mechanism(s) that will be used to manage records and data throughout the review	x	☐	350-360
Selection process	11 b	State the process that will be used for selecting studies (e.g., two independent reviewers) through each phase of the review (i.e., screening, eligibility, and inclusion in meta-analysis)	x	☐	365-380
Data collection process	11 c	Describe planned method of extracting data from reports (e.g., piloting forms, done independently, in duplicate), any processes for obtaining and confirming data from investigators	x	☐	390-410

Section/topic	#	Checklist item	Information reported		Line number(s)
			Yes	No	
**Data items**	12	List and define all variables for which data will be sought (e.g., PICO items, funding sources), any pre-planned data assumptions and simplifications	x	☐	410-430
**Outcomes and prioritization**	13	List and define all outcomes for which data will be sought, including prioritization of main and additional outcomes, with rationale	x	☐	420-430
**Risk of bias in individual studies**	14	Describe anticipated methods for assessing risk of bias of individual studies, including whether this will be done at the outcome or study level, or both; state how this information will be used in data synthesis	x	☐	N/A. Not applicable for scoping reviews as per JBI guidance
DATA					
**Synthesis**	15 a	Describe criteria under which study data will be quantitatively synthesized	x	☐	435-450
	15 b	If data are appropriate for quantitative synthesis, describe planned summary measures, methods of handling data, and methods of combining data from studies, including any planned exploration of consistency (e.g., I^2, Kendall's tau)	x	☐	N/A. Scoping review methodology does not include quantitative synthesis (JBI, PRISMA-ScR).
	15 c	Describe any proposed additional analyses (e.g., sensitivity or subgroup analyses, meta-regression)	x	☐	N/A. No additional analyses planned as per scoping review methodology (see Methods, lines 435-465)
	15 d	If quantitative synthesis is not appropriate, describe the type of summary planned	x	☐	N/A. Quantitative synthesis not appropriate; results will be synthesized descriptively and thematically according to JBI and PRISMA-ScR

Section/topic	#	Checklist item	Information reported		Line number(s)
			Yes	No	
					guidelines (see Methods, lines 435–465)
**Meta-bias(es)**	16	Specify any planned assessment of meta-bias(es) (e.g., publication bias across studies, selective reporting within studies)	x	☐	N/A. No assessment of meta-biases conducted as per scoping review methodology (see Methods, lines 435–465)
**Confidence in cumulative evidence**	17	Describe how the strength of the body of evidence will be assessed (e.g., GRADE)	x	☐	N/A. Not applicable as scoping reviews do not typically assess strength of evidence (per JBI and PRISMA-ScR).
