# Supplementary material for: Strategic communication to strengthen nurses’ professional identity and drive cultural change in healthcare organizations: A JBI scoping review protocol
Source: PLoS One. 2026 Sep 29;21(9):e0359546. doi: 10.1371/journal.pone.0359546 (PMC13623099; doi:10.1371/journal.pone.0359546)
Supplement: Supplementary file 5 [file pone.0359546.s005.pdf]

S5 Appendix. Tentative scoping review timeline

This timeline is indicative and may be adapted depending on the complexity of the literature, team availability, and unforeseen challenges.

Stage	Description	Estimated duration	Month	Responsible(s)
Phase 1: Preparation				
1.1. Protocol Development	Defining objectives, eligibility criteria, and search strategy.	4 weeks	1	CAJ, HGL, Lib.
1.2. Protocol Registration	Formal registration on Open Science Framework (OSF).	1 week	1	CAJ
Phase 2: Search and Selection				
2.1. Database Search	Executing search strategies across 7 databases and grey literature.	2 weeks	2	CAJ
2.2. Reference Management	Importing results into Covidence and removing duplicates.	1 week	2	CAJ
2.3. Title/Abstract Screening	Independent screening by two reviewers (blinded). Conflict resolution.	8-10 weeks	2-4	All authors
2.4. Full-Text Screening	Retrieval and independent screening of eligible full texts.	8 weeks	5-6	CAJ, all authors
Phase 3: Extraction and Analysis				
3.1. Tool Calibration	Final calibration and piloting of the data extraction tool.	1 week	7	All authors
3.2. Data Extraction	Independent extraction from included studies using standardized tool.	8-10 weeks	7-9	CAJ, all authors
3.3. Data Analysis	Quantitative descriptive analysis and thematic qualitative analysis. Glossary development.	6-8 weeks	9-11	CAJ, all authors

3.4. Convergent Synthesis	Integration of findings and mapping to analytical frameworks. Development of conceptual models/diagrams.	3-5 weeks	11-12	CAJ, all authors
Phase 4: Dissemination				
4.1. Expert Consultation	Post-synthesis consultation for validation.	2–3 weeks	12	Team review and External expert
4.2. Manuscript Drafting	Drafting results, discussion, and conclusion sections.	6 weeks	12-14	CAJ, all authors
4.3. Internal Review	Critical review by co-authors and final editing.	3 weeks	14	All authors
4.4. Submission	Formatting, reference checking, and journal submission.	1–2 weeks	14-15	All authors

Note. After title and abstract screening, full-text screening, data extraction, and analysis are conducted sequentially, with initial steps performed by the doctoral candidate (CAJ), followed by independent team review and consensus.

Abbreviations. CAJ: Carmen Arteaga-Jordá; HGL: Hildegart González-Luis; MJYG: María Jesús Yáñez-Galdames; MJHL: María José Hernández-Leal; COO: Cristina Oroviogoicoechea Ortega; Lib.: Librarian.
