# Supplementary material for: Strategic communication to strengthen nurses’ professional identity and drive cultural change in healthcare organizations: A JBI scoping review protocol
Source: PLoS One. 2026 Sep 29;21(9):e0359546. doi: 10.1371/journal.pone.0359546 (PMC13623099; doi:10.1371/journal.pone.0359546)
Supplement: Supplementary file 6 [file pone.0359546.s006.pdf]

S6 Appendix. Search Strategy

Database: MEDLINE (via PubMed – NCBI)

Date of search: 22nd July 2025

Limits applied: Language – none; Date – January 2000 to July 2025

Number of results retrieved: 1,625 records

Search string:

("nurse s role"[MeSH Terms] OR "nurse s role"[Title/Abstract] OR "nurses"[MeSH Terms] OR "nurses"[Title/Abstract] OR "professional practice model"[Title/Abstract] OR "professional nursing practice model"[Title/Abstract] OR "professional identity"[Title/Abstract] OR "professional practice"[Title/Abstract] OR "nursing models"[Title/Abstract] OR "models, nursing"[MeSH Terms]) AND ("organizational culture"[MeSH Terms] OR "organizational culture"[Title/Abstract] OR "cultural transformation"[Title/Abstract] OR "models, organizational"[MeSH Terms] OR "organizational models"[Title/Abstract] OR "change management"[Title/Abstract] OR "change management"[MeSH Terms] OR "organizational innovation"[MeSH Terms] OR "organizational innovation"[Title/Abstract]) AND ("communication"[MeSH Terms] OR "communication"[Title/Abstract] OR "organizational communication"[Title/Abstract] OR "strategic communication"[Title/Abstract] OR "strategic planning"[Title/Abstract] OR "strategic planning"[MeSH Terms]) AND 2000/01/01:2025/12/31[Date - Publication]) AND (2000:2025[pdat])

Validation:

The search strategy was developed in collaboration with a university librarian and peer-reviewed using the PRESS (Peer Review of Electronic Search Strategies) checklist. The full PRESS documentation, including validation worksheets, has been published openly in the Open Science Framework (OSF): https://doi.org/10.17605/OSF.IO/VHC4K
