# Supplementary material for: Strategic communication to strengthen nurses’ professional identity and drive cultural change in healthcare organizations: A JBI scoping review protocol
Source: PLoS One. 2026 Sep 29;21(9):e0359546. doi: 10.1371/journal.pone.0359546 (PMC13623099; doi:10.1371/journal.pone.0359546)
Supplement: Supplementary file 7 [file pone.0359546.s007.pdf]

S7 Appendix. Database Search Adaptation Framework

Database	Adaptation approach	Search string
**CINAHL**	CINAHL Headings + TI/AB	(MH "Strategic Planning" OR MH "Communication" OR XB "strategic planning" OR XB "organizational communication" OR XB "strategic communication" OR XB "communication") AND (MH "Organizational Culture" or MH "Change Management" or XB "organizational culture" or XB "cultural transformation" or XB "organizational models" or XB "change management") AND (MH "Professional Identity" OR MH "Nursing Role" OR XB "Professional Identity" OR XB "Nursing Role" OR XB "professional nursing practice model" XB "professional nursing practice model" OR MH "Nursing Models, Theoretical" OR XB "Nursing Models") 249 Hits--> Limit to yr="2000 - 2025"
**WoS**	TI/AB	(TI=("strategic planning" OR "organizational communication" OR "strategic communication" OR "communication")) OR AB=(("strategic planning" OR "organizational communication" OR "strategic communication" OR "communication")) AND (AB=("Organizational Culture" or "Change Management" or "cultural transformation" or "organizational models")) OR TI=("Organizational Culture" or "Change Management" or "cultural transformation" or "organizational models") AND (TI=("Professional Identity" OR "Nursing Role" OR "professional nursing practice model" OR "Nursing Models")) OR AB=(("Professional Identity" OR "Nursing Role" OR "professional nursing practice model" OR "Nursing Models")) Limit to yr="2000 - 2025"

**Scopus**	TITLE-ABS	((TITLE-ABS-KEY ("nurse s role" OR "nurses" OR "professional practice model" OR "professional nursing practice model" OR "professional identity" OR "professional practice" OR "nursing models") AND TITLE-ABS-KEY ("organizational culture" OR "cultural transformation" OR "organizational models" OR "change management" OR "organizational innovation") AND TITLE-ABS-KEY ("organizational culture" OR "cultural transformation" OR "organizational models" OR "change management" OR "organizational innovation") AND TITLE-ABS-KEY ("communication" OR "organizational communication" OR "strategic communication" OR "strategic planning")) AND PUBYEAR > 1999) AND ORIG-LOAD-DATE > 1771504527
**PsyqInfo**	PsyqInfo Headings + TI/AB	(MH "Strategic Planning" OR MH "Communication" OR XB "strategic planning" OR XB "organizational communication" OR XB "strategic communication" OR XB "communication") AND (MH "Organizational Culture" or MH "Change Management" or XB "organizational culture" or XB "cultural transformation" or XB "organizational models" or XB "change management") AND (MH "Professional Identity" OR MH "Nursing Role" OR XB "Professional Identity" OR XB "Nursing Role" OR XB "professional nursing practice model" XB "professional nursing practice model" OR MH "Nursing Models, Theoretical" OR XB "Nursing Models") 249 Hits--> Limit to yr="2000 - 2025"
**Dialnet**	Spanish free-text adaptation	identidad profesional enfermera, comunicación estratégica, cambio cultural. Filtro año: 2000–2025
**SciELO**	Spanish free-text adaptation	identidad profesional enfermera, comunicación estratégica, cambio cultural. Filtro año: 2000–2025
