# Supplementary material for: Strategic communication to strengthen nurses’ professional identity and drive cultural change in healthcare organizations: A JBI scoping review protocol
Source: PLoS One. 2026 Sep 29;21(9):e0359546. doi: 10.1371/journal.pone.0359546 (PMC13623099; doi:10.1371/journal.pone.0359546)
Supplement: Supplementary file 8 [file pone.0359546.s008.pdf]

S8 Appendix. Data Extraction Instrument and Coding Guide

Integrated Data Extraction Tool

Field	Description / Coding Instructions
Citation	Author(s), year, title, journal
Study design / Source type	Qualitative, quantitative, mixed methods, conceptual, opinion, grey literature, etc.
Setting/Type	Hospital, clinic, primary care, long-term care, etc.
Organizational level	Micro (unit), meso (hospital), macro (system)
Sector	Public, private, not-for-profit
Country	Country or region where the study was conducted
Phase of change	Is the organization undergoing, planning, or recently completed a process of cultural/professional transformation? (Planning / Implementation / Consolidation / Not reported. Specify if linked to any excellence model or accreditation process.)
Excellence model type / Accreditation status	Magnet® Recognition Program, Pathway to Excellence®, other excellence model (specify), no formal model, not reported
Participants	Who was included (roles, numbers, professional category, etc.)
Main conceptual focus	What is the primary conceptual focus of the study? (e.g. strategic communication and cultural change, strategic communication and professional identity, strategic communication only, or a combination of all three concepts)

Field	Description / Coding Instructions
Methods	Data collection and analysis methods (e.g. interviews, surveys, document analysis, etc.)
Professional Identity	ISPIN 1 | ISPIN 2 | ISPIN 3 | ISPIN 4
Communication	Which of the 14 strategic communication components are addressed? (List numbers: C. Comp. 1 -14)
Cultural transformation	Which of the 8 dimensions are present? Code as ENC (enculturation), LED (leadership), GOV (governance), LEA (learning), SAF (safety), DEI (diversity), IMP (implementation), OUT (outcomes). with values 0 = absent, 1 = in process, 2 = consolidated.

Source: Own elaboration based on a synthesis of relevant literature and analytical judgment. Adapted in part from: Aromataris E, Munn Z (Editors). JBI Manual for Evidence Synthesis. Joanna Briggs Institute; 2020. Retrieved from: https://synthesismanual.jbi.global

Note: References for this section are provided in S9 Appendix: References for Supporting Information.

Illustrative Example of Instrument Application

The following fictitious study is provided solely for illustrative purposes to demonstrate the application of the data extraction instrument and coding framework. It does not correspond to any study included in the review.

Field	Example Entry
**Citation**	Taylor J, Smith R. (2022). Strategic communication and nursing engagement during organizational transformation. *PLOS ONE*.
**Study design / Source type**	Mixed methods
**Setting/Type**	Acute care hospital
**Organizational level**	Meso (hospital)
**Sector**	Public
**Country**	Australia
**Phase of change**	Implementation – organizational transformation initiative focused on nursing professional practice
**Excellence model type / Accreditation status**	No formal model reported
**Participants**	Registered nurses (n=150), nurse managers (n=20)
**Main conceptual focus**	Strategic communication, cultural transformation, and professional identity
**Methods**	Surveys, focus groups, and document analysis
**Professional Identity**	ISPIN 2; ISPIN 3; ISPIN 4
**Communication**	Components 2, 5, 8, 11
**Cultural transformation**	LED, GOV, IMP

Note. This example is intended solely to illustrate completion of the data extraction instrument. All study details and coding entries are fictitious and provided for demonstration purposes only.
