# Supplementary material for: Strategic communication to strengthen nurses’ professional identity and drive cultural change in healthcare organizations: A JBI scoping review protocol
Source: PLoS One. 2026 Sep 29;21(9):e0359546. doi: 10.1371/journal.pone.0359546 (PMC13623099; doi:10.1371/journal.pone.0359546)
Supplement: Supplementary file 9 [file pone.0359546.s009.pdf]

S9 Appendix. References for Supporting Information

1. Rogers EM. Diffusion of innovations [book]. 5th ed. New York: Free Press; 2003.

2. Kreps GL. Evaluating health communication programs to enhance health care and health promotion. J Health Commun. 2014 Dec 2;19(12):1449–59. doi:10.1080/10810730.2014.954080 PubMed PMID: 25491580.

3. Cornelissen J. Corporate communication : a guide to theory and practice [book]. 6th ed. Los Angeles [etc: SAGE; 2020.

4. Jo Hatch M, Schultz M. Relations between organizational culture, identity and image [article]. Balmer JMT, van Riel CBM, editors. Eur J Mark. 1997;31(5/6):356–65. doi:10.1108/eb060636

5. Joseph ML, Godfrey N. A New Mindset: From Tasks to Professional Identity. Nurse Lead. 2023 Apr 1;21(2):183–7. doi:10.1016/J.MNL.2022.12.007

6. Argenti PA. Corporate communication [book]. 8th ed. Boston: McGraw-Hill Irwin; 2023.

7. Barrett DJ. Leadership Communication: A Communication Approach for Senior-Level Managers. Handb Bus Strategy [Internet]. Emerald Group Publishing Limited; 2006 [cited 2025 Jun 27]. Available from: https://hdl.handle.net/1911/27037

8. Glanz K, Rimer BK. Theory at a glance a guide for health promotion practice. 2nd ed. [Bethesda, MD] : U.S. Dept. of Health and Human Services, National Institutes of Health, National Cancer Institute,; 2005.

9. World Health Organization. WHO I Communicating for health: Strategic communications framework for effective communications. [Internet]. 2017 [cited 2025 Jun 27]. Available from: https://www.who.int/about/communications

10. Valente TW. Social Networks and Health: Models, Methods, and Applications. Social Networks and Health: Models, Methods, and Applications [Internet]. Oxford University Press; 2010 [cited 2025 Jun 27]. 1–296 p. Available from: https://academic.oup.com/book/4172 doi:10.1093/ACPROF:OSO/9780195301014.001.0001

11. American Nurses Credentialing Center. 2023 Magnet® Application Manual. Silver Spring, MD; 2023.

12. Goodman MB. Corporate communication : strategic adaptation for global practice [book]. Hirsch PB, editor. New York: Lang; 2010.

13. Brewington J, Godfrey N. The Professional Identity in Nursing Initiative. Nurs Educ Perspect. 2020 May 1;41(3):201. doi:10.1097/01.NEP.0000000000000667 PubMed PMID: 32310908.

14. Benner PE. From novice to expert : excellence and power in clinical nursing practice [book]. Menlo Park, California: Addison-Wesley; 1984.

15. Maanen J Van, Schein EH. Toward a theory of organizational socialization. In: Staw BM, editor. Research in Organizational Behavior (Vol. 1) [Internet]. Greenwich, CT: JAI Press; 1979 [cited 2025 Sep 16]. p. 209–64. Available from: https://dspace.mit.edu/handle/1721.1/1934

16. Kotter JP. Leading change [book]. Boston: Harvard Business School Press; 1996.

17. Bass BM, Riggio RE. Transformational leadership. Transformational Leadership: Second Edition [Internet]. Second edition. New York: Lawrence Erlbaum Associates; 2006 [cited 2025 Sep 16]. 296 p. Available from: https://www.taylorfrancis.com/books/mono/10.4324/9781410617095/transformational-leadership-bernard-bass-ronald-riggio doi:10.4324/9781410617095/TRANSFORMATIONAL-LEADERSHIP-BERNARD-BASS-RONALD-RIGGIO/RIGHTS-AND-PERMISSIONS

18. Kanter RM. Men and Women of the Corporation [Internet]. New York: Basic Books; 1977 [cited 2025 Sep 16]. Available from: https://www.hbs.edu/faculty/Pages/item.aspx?num=10807

19. Laschinger HKS. A theoretical approach to studying work empowerment in nursing: a review of studies testing Kanter's theory of structural power in organizations. Nurs Adm Q. 1996;20(2):25–41. doi:10.1097/00006216-199602020-00006 PubMed PMID: 8700383.

20. Kramer M, Schmalenberg C, Maguire P. Essentials of a magnetic work environment: Part 1. Nursing (Brux). 2004 Jun;34(6):50–4. doi:10.1097/01.NURSE.0000304729.97476.61

21. Argyris C, Schön DA. Organizational Learning II Theory, Method and Practice. [Internet]. 1st ed. (FAC SUB). Reading, MA: Addison-Wesley; 1996 [cited 2025 Sep 16]. 336. Available from: https://www.scirp.org/reference/referencespapers?referenceid=1423922

22. Senge P. The fifth discipline: The art and practice of the learning organization. Human Resource Management [Internet]. New York: Currency Doubleday; 1990 [cited 2025 Sep 16]. 371. Available from: https://www.e-education.psu.edu/geog468/sites/www.e-education.psu.edu.geog468/files/TheFifthDiscipline.pdf doi:10.1002/HRM.3930290308

23. Greenhalgh T, Robert G, Macfarlane F, Bate P, Kyriakidou O. Diffusion of Innovations in Service Organizations: Systematic Review and Recommendations. Milbank Q. 2004 Dec 1;82(4):581–629. doi:10.1111/J.0887-378X.2004.00325.X PubMed PMID: 15595944.

24. Agency for Healthcare Research and Quality. Hospital Survey on Patient Safety Culture (SOPS®) [Internet]. Rockville, MD; 2004 [cited 2025 Sep 16]. Available from: https://www.ahrq.gov/sops/surveys/hospital/index.html

25. Reason JT. Managing the risks of organizational accidents : Reason, James T. : Free Download, Borrow, and Streaming : Internet Archive [Internet]. Aldershot, Hants, England; Brookfield, VT, USA: Ashgate; 1997 [cited 2025 Sep 16]. xvii–252. Available from: https://archive.org/details/managingrisksofo0000reas

26. Campinha-Bacote J. The Process of Cultural Competence in the Delivery of Healthcare Services: A Model of Care. Journal of Transcultural Nursing. 2002 Jun;13(3):181–4. doi:10.1177/10459602013003003 PubMed PMID: 12113146.

27. Leininger MM. Culture care diversity and universality : a theory of nursing [Internet]. New York : National League for Nursing Press; 1991 [cited 2025 Sep 16]. 432 p. Available from: https://archive.org/details/culturecarediver00lein

28. Consolidated Framework for Implementation Research (CFIR). Consolidated Framework for Implementation Research [Internet]. [cited 2025 Sep 16]. Technical Assistance for users of the CFIR framework. Available from: https://cfirguide.org/

29. Fixsen DL, Blase KA, Naoom SF, Wallace F. Implementation research: aSynthesis of the literature [Internet]. Tampa, FL; 2005 [cited 2025 Sep 16]. Available from: https://nirn.fpg.unc.edu/sites/nirn.fpg.unc.edu/files/resources/NIRN-MonographFull-01-2005.pdf

30. Aiken LH, Sloane DM, Bruyneel L, Van Den Heede K, Griffiths P, Busse R, et al. Nurse staffing and education and hospital mortality in nine European countries: a retrospective observational study. The Lancet. 2014 May 24;383(9931):1824–30. doi:10.1016/S0140-6736(13)62631-8 PubMed PMID: 24581683.

31. Davies HTO, Nutley SM, Mannion R. Organisational culture and quality of health care. BMJ Qual Saf. 2000 Jun 1;9(2):111–9. doi:10.1136/QHC.9.2.111 PubMed PMID: 11067249.

32. Donabedian A. An introduction to quality assurance in health care. Croatian Medical Journal. New York, NY: Oxford University Press; 2003. xiii–200.

33. Shamseer L, Moher D, Clarke M, Ghersi D, Liberati A, Petticrew M, et al. Preferred reporting items for systematic review and meta-analysis protocols (PRISMA-P) 2015: elaboration and explanation. BMJ. 2015 Jan 2;349. doi:10.1136/BMJ.G7647 PubMed PMID: 25555855.
