# Supplementary material for: Strategic communication to strengthen nurses’ professional identity and drive cultural change in healthcare organizations: A JBI scoping review protocol
Source: PLoS One. 2026 Sep 29;21(9):e0359546. doi: 10.1371/journal.pone.0359546 (PMC13623099; doi:10.1371/journal.pone.0359546)
Supplement: Supplementary file 10 [file pone.0359546.s010.pdf]

Consolidated Analysis Report of the Scoping Review Protocol

I. General Observations

General Strengths:

- **Methodological Rigor:** Strong adherence to JBI methodology for scoping reviews and commitment to PRISMA-ScR reporting guidelines, ensuring transparency, reproducibility, and quality of the review.
- **In-depth Conceptualization:** The proposal to utilize three complementary analytical frameworks (a 14-component strategic communication framework, a 4-domain ISPIN-IBIN model for professional identity, and an 8-dimension cultural transformation framework) to guide study selection and data extraction is a major differentiating factor, enabling a multifaceted and rich analysis of the phenomenon under study.
- **Transparency and Accountability:** The registration of the protocol on the Open Science Framework (OSF) with a specific DOI enhances the transparency of the process and the accountability of the authors.
- **Comprehensive Search:** The search strategy will be conducted across seven bibliographic databases and complemented by grey literature searches, with no language restrictions, maximizing the retrieval of relevant literature.

General Weaknesses:

- **Absence of Detailed Timeline:** The lack of an explicit timeline for the review phases may hinder project planning and progress monitoring.
- **Post-Synthesis Expert Consultation:** While expert consultation was conducted during the initial phases, the absence of a formal post-synthesis stakeholder consultation step to validate preliminary findings and refine recommendations represents a missed opportunity to enrich the results and enhance their practical applicability.

General Suggestions for Improvement:

- **Incorporate an Explicit Timeline:** Adding a detailed timeline for all review phases would aid project management and communication of progress.
- **Consider Expert Consultation for Validation:** Evaluate the inclusion of a formal expert or stakeholder consultation step (such as the optional fifth stage of the JBI methodology for scoping reviews) to validate preliminary results and the practical implications of the review.

II. Analysis by Specific Manuscript Sections/Points

1. Presentation of Results

- **Strengths:**
    - **Adequate and Detailed Plan:** The protocol presents a clear and multifaceted preliminary plan for the presentation of results, which is **ADEQUATE**. The authors specify that they will use:

        - **Tables and Figures:** To summarize quantitative data (such as publication year, country, study design, setting), as indicated in *PONE-D-25-53788_reviewer, Page 12*: "Quantitative data (e.g., publication year, country, study design, setting) will be summarized in tables and figures to map the evidence."
        - **Conceptual Models or Evidence-Mapping Diagrams:** To illustrate relationships between communication strategies, contextual factors, and outcomes. *PONE-D-25-53788_reviewer, Page 12*, states: "Conceptual models or evidence-mapping diagrams may be developed to illustrate relationships among communication strategies, contextual factors, and outcomes."
        - **Descriptive Tables and Narrative Summaries:** To systematically map the results to the three analytical frameworks (the 14-component strategic communication model, the four-domain ISPIN-IBIN framework, and the eight-dimension cultural transformation framework). As per *PONE-D-25-53788_reviewer, Page 13*: "Results will be presented in descriptive tables and narrative summaries, systematically mapped to the 14-component strategic communication model, the four-domain ISPIN-IBIN framework, and the eight-dimension cultural transformation framework."
    - **Clarity and Structure:** The manner in which results will be presented is well-articulated, directly linking to the operational frameworks that will guide the analysis.
- **Weaknesses:**
    - No weaknesses were identified in this aspect, as the presentation plan is comprehensive and well-described.
- **Suggestions for Improvement:**
    - No additional suggestions are needed for this point, as the current description is robust and well-planned. Consistent implementation of this plan will be crucial.

2. Expert Consultation

- **Strengths:**
    - **Robust Initial Consultation:** The team demonstrated **ADEQUACY** by conducting expert consultations during crucial phases of protocol development, which is a strong point.
        - The 14-component strategic communication framework was "refined through expert consultation and pilot testing" (*PONE-D-25-53788_reviewer, Page 6*).
        - The search strategy was "developed with a university librarian and peer-reviewed via PRESS" (*PONE-D-25-53788_reviewer, Page 11*), and specific acknowledgment is given to Professor Richard Ricciardi for

"expert insights and critical discussion of the topic, as well as his feedback on the preliminary search strategy" (*PONE-D-25-53788_reviewer, Page 17*).

  - **Dissemination Plan for Stakeholders:** There is a plan for "stakeholder briefings for nursing leadership, organizational development, and health communication communities" (*PONE-D-25-53788_reviewer, Page 16*), ensuring that findings will reach the relevant audience.

- **Weaknesses:**
  - **Absence of Formal Post-Synthesis Consultation:** The main weakness is the absence of a formal and explicit plan for an expert or stakeholder consultation step as an *additional stage of the scoping review itself*, specifically to validate or refine preliminary findings and recommendations. JBI methodology for scoping reviews often includes this optional stage (the fifth stage) to enhance the relevance and applicability of the findings. The consultation mentioned in the protocol occurred *before* the review (protocol development) or is planned for *after* the review's completion (dissemination).

- **Suggestions for Improvement:**
  - **Include a Formal Post-Synthesis Consultation Step:** It is recommended to consider including a formal expert or stakeholder consultation step *after the preliminary synthesis of results*. This step could involve presenting the findings to a panel of experts (nurses, healthcare leaders, communication specialists) to gather feedback on data interpretation, identify additional gaps, and validate the relevance and practicality of the implications and recommendations. This would further align the protocol with best practices for scoping reviews and enhance the impact of the results.

3. Protocol Registration

- **Strengths:**
  - **Appropriate Registration:** The protocol was **ADEQUATELY** registered, which is a point of research excellence.
  - **Transparency and Reproducibility:** Registration on the Open Science Framework (OSF) with a specific DOI (Digital Object Identifier) strongly indicates a commitment to research transparency and reproducibility. As per *PONE-D-25-53788_reviewer, Page 9*: "The protocol is registered on the Open Science Framework, with any amendments documented publicly [38]." And in *PONE-D-25-53788_reviewer, Page 16*: "The protocol has been prospectively registered on the Open Science Framework (OSF): https://doi.org/10.17605/OSF.IO/VHC4K".

- **Weaknesses:**
  - No weaknesses identified.

- **Suggestions for Improvement:**
  - No suggestions are necessary, as this aspect is excellently addressed.

4. PRISMA-ScR Compliance Statement

- **Strengths:**
  - **Clear Commitment to Guidelines:** The compliance statement is **ADEQUATE** and explicit.
  - **Reporting Quality and Transparency:** The clear mention that the review will follow PRISMA-ScR (Preferred Reporting Items for Systematic reviews and Meta-Analyses extension for Scoping Reviews) guidelines is a strong point. This ensures that the final report will be comprehensive, transparent, and of high quality, facilitating its interpretation and application by other researchers and professionals. In *PONE-D-25-53788_reviewer, Page 9*: "This review will follow the Joanna Briggs Institute (JBI) methodology for scoping reviews [35,36] and report according to PRISMA-ScR [37]." And reinforced in *PONE-D-25-53788_reviewer, Page 15*: "Adherence to established methodological standards (JBI framework, PRISMA-ScR guidelines)."
- **Weaknesses:**
  - No weaknesses identified.
- **Suggestions for Improvement:**
  - No suggestions are necessary, as this aspect is well addressed.

5. Timeline

- **Strengths:**
  - No specific strengths related to a timeline were identified, as the protocol does not include one.
- **Weaknesses:**
  - **Absence of Explicit Timeline:** The primary weakness is that the protocol **DOES NOT** include an explicit or detailed tentative timeline for the completion of the scoping review. While it mentions the cutoff date for the literature to be searched (2000 to 2025), this refers to the period of literature inclusion, not the execution time of the review project itself. The lack of estimated phases (search, screening, extraction, analysis, writing, etc.) can hinder planning and time management.
- **Suggestions for Improvement:**
  - **Add a Detailed Timeline:** It is **essential** to add a detailed tentative timeline specifying the estimated durations for each phase of the review. This not only aids in project planning and execution but also enhances the transparency of the protocol. Below, I provide a timeline template that could be adapted and included:

Tentative Scoping Review Timeline Template

This timeline is an estimate and may be adjusted based on the complexity of the literature, team availability, and unexpected challenges that may arise.

Stage	Description	Estimated Duration	Suggested Period (Example)	Responsible(s)
**Phase 1: Preparation**				
1.1. Protocol Development & Search Strategy Refinement	Defining objectives, review questions, eligibility criteria, and detailed search strategy. (Already completed, but important for formal time recording).	4 weeks	Month 1	Carmen Arteaga-Jordá (CAJ), Hildegart González-Luis (HGL), Librarian
1.2. Protocol Registration	Formal registration of the protocol on a platform like OSF. (Already completed).	1 week	Month 1	CAJ, HGL
**Phase 2: Search and Selection**				
2.1. Database Search Execution	Executing comprehensive search strategies across seven bibliographic databases and grey literature.	2 weeks	Month 2	CAJ
2.2. Reference Management & Deduplication	Importing all search results into Covidence and removing duplicates.	1 week	Month 2	CAJ
2.3. Title and Abstract Screening	Independent screening of titles and abstracts by two reviewers (blinded). Conflict resolution.	4-6 weeks	Month 3 - Month 4	CAJ, María Jesús Yáñez-Galdames (MJYG) (and HGL for conflicts)
2.4. Full-Text Screening	Retrieval and independent full-text screening of eligible studies. Documentation of exclusion reasons. Conflict resolution.	4-6 weeks	Month 4 - Month 5	CAJ, MJYG (and HGL for conflicts)
**Phase 3: Extraction and Analysis**				

Stage	*Description*	*Estimated Duration*	*Suggested Period (Example)*	*Responsible(s)*
3.1. Data Extraction Tool Calibration	*Conducting final calibration and piloting of the data extraction tool (if necessary, beyond initial piloting).*	*1 week*	*Month 6*	*CAJ, MJYG*
3.2. Data Extraction	*Independent data extraction from included studies using the standardized tool. Contacting authors for missing information.*	*6-8 weeks*	*Month 6 - Month 7*	*CAJ, María José Hernández-Leal (MJHL)*
3.3. Data Analysis (Quantitative & Qualitative)	*Descriptive analysis of quantitative data and thematic analysis of qualitative data. Glossary development.*	*4-6 weeks*	*Month 8 - Month 9*	*CAJ, HGL, Cristina Oroviogoicoechea Ortega (COO)*
3.4. Convergent Synthesis	*Integration of quantitative and qualitative findings, mapping them to the frameworks. Development of conceptual models/diagrams.*	*3 weeks*	*Month 9 - Month 10*	*CAJ, HGL, COO*
Phase 4: Dissemination				
4.1. Manuscript Drafting	*Drafting the initial manuscript of the scoping review, including results, discussion, and conclusion sections.*	*6 weeks*	*Month 10 - Month 11*	*CAJ (initial draft), All authors (review & editing)*
4.2. Internal Review and Feedback	*Critical review of the manuscript by co-authors and incorporation of feedback.*	*3 weeks*	*Month 12*	*All authors*
4.3. Preparation for Submission	*Formatting, reference checking, preparation of figures and tables, etc.*	*1 week*	*Month 12 - Month 13*	*CAJ*

Stage	*Description*	*Estimated Duration*	*Suggested Period (Example)*	*Responsible(s)*
4.4. Journal Submission & Post-Submission Revisions	*Submission of the manuscript to a peer-reviewed journal and response to reviewers.*	*Variable (3-6 months or more)*	*From Month 13 onwards*	*All authors*
4.5. Presentations and Stakeholder Briefings	*Preparation and delivery of conference presentations and briefings for stakeholders.*	*Ongoing*	*From Month 13 onwards*	*All authors*

6. Funding

- **Strengths:**
    - **Full Transparency:** Funding declarations are **ADEQUATE** and provided clearly and explicitly in two sections of the protocol. In *PONE-D-25-53788_reviewer, Page 2*, under "Financial Disclosure," it states: "The author(s) received no specific funding for this work." And again, in *PONE-D-25-53788_reviewer, Page 16*, under the "Funding" section: "This scoping review did not receive any specific grant from funding agencies in the public, commercial, or not-for-profit sectors." This duplication reinforces the clarity of the information.
- **Weaknesses:**
    - No weaknesses identified.
- **Suggestions for Improvement:**
    - No suggestions are necessary, as this aspect is perfectly addressed.

7. Conflicts of Interest

- **Strengths:**
    - **Clear Declaration:** The declaration of potential conflicts of interest is **ADEQUATE** and transparent. Similar to funding, the information is provided in two sections of the protocol, ensuring no ambiguity. In *PONE-D-25-53788_reviewer, Page 2*, under the "Competing Interests" section, the authors declare: "The authors have declared that no competing interests exist." And in *PONE-D-25-53788_reviewer, Page 16*, under the "Conflicts of interest" section: "The authors declare no competing interests."
- **Weaknesses:**
    - No weaknesses identified.
- **Suggestions for Improvement:**

- No suggestions are necessary, as this aspect is very well addressed.

8. Appendices: Detailed Search Strategy

- **Strengths:**
  - **Adequate Planning:** The protocol demonstrates **ADEQUACY** by planning to include the detailed search strategy as an appendix.
  - **Search Reproducibility:** The clear mention that the "detailed search strategy for at least one major database" will be included in Appendix S5 is crucial for the review's reproducibility. In *PONE-D-25-53788_reviewer, Page 10*, the "Search strategy" section refers to "(S5 Appendix)," and *PONE-D-25-53788_reviewer, Page 23*, specifies: "S5 Appendix. Full search strategy. Complete database search strings, PRESS peer-review documentation, and OSF DOI for protocol registration."
- **Weaknesses:**
  - No weaknesses identified.
- **Suggestions for Improvement:**
  - No suggestions are necessary, as this aspect is perfectly addressed in the planning.

9. Appendices: Data Extraction Instrument

- **Strengths:**
  - **Adequate Planning:** The protocol demonstrates **ADEQUACY** by planning to include the data extraction instrument as an appendix.
  - **Extraction Transparency:** The inclusion of the data extraction instrument (Appendix S6) is a strong point that promotes transparency and reproducibility of the extraction process. As per *PONE-D-25-53788_reviewer, Page 12*, in "Data extraction," the authors state: "(see S6 Appendix)." And *PONE-D-25-53788_reviewer, Page 23*, in "Supporting information captions," specifies: "S6 Appendix. Integrated data extraction tool. Standardized, piloted extraction form adapted from JBI methodology for charting study data."
- **Weaknesses:**
  - No weaknesses identified.
- **Suggestions for Improvement:**
  - No suggestions are necessary, as this aspect is perfectly addressed in the planning.
