# Supplementary material for: Strategic communication to strengthen nurses’ professional identity and drive cultural change in healthcare organizations: A JBI scoping review protocol
Source: PLoS One. 2026 Sep 29;21(9):e0359546. doi: 10.1371/journal.pone.0359546 (PMC13623099; doi:10.1371/journal.pone.0359546)
Supplement: Supplementary file 13 [file pone.0359546.s016.pdf]

Assessment of Author Revisions — Manuscript PONE-D-25-53788 (R2 → R3)

This report evaluates each recommendation made in our previous peer review of the scoping review protocol titled *"Strategic communication to strengthen nurses' professional identity and drive cultural change in healthcare organizations: A JBI Scoping Review Protocol"* (PONE-D-25-53788_R2), against the revised version submitted as PONE-D-25-53788_R3. Each recommendation is classified as **FULLY ADDRESSED**, **PARTIALLY ADDRESSED**, or **NOT ADDRESSED**. For items marked as partially addressed, specific remaining shortcomings are indicated.

Priority Recommendations (Mandatory)

Recommendation 1 — Full search strategy as appendix: Include the complete search string for MEDLINE/PubMed (or another primary database) with MeSH terms, Boolean operators, free-text keywords, applied filters, and number of results retrieved. S4 Appendix should be verified and, if absent, added.

Status: ✅ FULLY ADDRESSED

In the Response to Reviewers (Part 4 — Priority Recommendations), the authors state: *"We have added the complete search strategy for PubMed as S6 Appendix."* The manuscript confirms the inclusion of the full search strategy as a supplementary appendix, thereby meeting the requirement for transparency and reproducibility of the search process.

Recommendation 2 — Data extraction instrument as appendix: Make the complete data extraction form/template available as supplementary material (S5 Appendix), containing all fields to be extracted from each included study.

Status: ✅ FULLY ADDRESSED

The authors report: *"We have added the data extraction instrument as S5 Appendix."* The revised manuscript confirms the availability of the complete extraction instrument in appendix format, fulfilling the requirement for methodological transparency.

Recommendation 3 — Discussion of limitations and biases: Add a section or paragraph acknowledging the inherent limitations of the scoping review methodology (absence of critical quality appraisal, publication bias, language bias, subjectivity of narrative synthesis) and the mitigations adopted.

Status: ✅ FULLY ADDRESSED

The authors have incorporated a dedicated **"Strengths and Limitations"** section into the manuscript, which explicitly addresses:

- The absence of critical quality appraisal of included studies
- Potential publication bias, with overrepresentation of contexts with greater academic output (USA, Europe, Australia)
- Language bias, given that databases predominantly index English-language literature despite the absence of language restrictions
- Mitigations adopted: two independent reviewers, multiple databases, grey literature searching, and expert consultation

This section adequately covers all points raised in the original recommendation.

Secondary Recommendations

Recommendation 4 — Verification of all mentioned appendices (S1–S5) in the submission package.

Status: ✅ FULLY ADDRESSED

The authors confirm that all appendices (S1 through S6) have been included in the submission package. The updated appendix list is as follows:

- S1 Appendix: Strategic communication framework
- S2 Appendix: ISPIN-IBIN nursing professional identity model
- S3 Appendix: Eight-dimension cultural transformation framework
- S4 Appendix: Strategic communication framework (14 components)
- S5 Appendix: Data extraction instrument
- S6 Appendix: Complete search strategy for PubMed

Recommendation 5 — Detail the expert consultation plan: Specify the estimated number of experts, selection criteria, and consultation method (e.g., Delphi technique, interviews, focus group).

Status: ✖ NOT ADDRESSED

The **Expert Consultation** section remains unchanged from the previous version, retaining the same generic wording. The following elements have not been specified:

- Estimated number of experts to be consulted
- Selection criteria (e.g., years of experience, area of specialization, academic output)
- Consultation method (e.g., Delphi technique, semi-structured interviews, focus group, online questionnaire)

Required action: The authors should elaborate on the expert consultation plan by providing at least the estimated number of participants, the inclusion criteria (e.g., minimum of five years of experience in strategic health communication, nursing, or organizational change), and the data collection method (e.g., two-round Delphi technique, semi-structured interviews, or online focus group).

Additional Items from the Review Report (Section 1.4 — Management of Bias)

The recommendation concerning **Management of Bias** has been fully absorbed by the newly added **Strengths and Limitations** section, which now covers all requested points, including publication bias, language bias, the absence of critical quality appraisal, and the mitigations adopted. This item is therefore considered **FULLY ADDRESSED**.

Summary of Compliance Rate

Recommendation	Status
1. Full search strategy as appendix	✅ FULLY ADDRESSED
2. Data extraction instrument as appendix	✅ FULLY ADDRESSED
3. Discussion of limitations and biases	✅ FULLY ADDRESSED
4. Verification of all appendices in submission package	✅ FULLY ADDRESSED
5. Detailed expert consultation plan	❌ NOT ADDRESSED

Overall Compliance Rate: 80% (4 out of 5 recommendations addressed)

Final Assessment

Manuscript PONE-D-25-53788_R3 has satisfactorily addressed all mandatory (priority) recommendations from the previous review round. The complete search strategy for PubMed has been included as a supplementary appendix, the data extraction instrument has been made available, and a dedicated discussion of methodological limitations and biases has been incorporated into the manuscript. These changes substantially enhance the transparency, reproducibility, and methodological rigor of the protocol.

The sole outstanding recommendation — detailing the expert consultation plan — is a secondary recommendation that does not compromise the methodological validity of the protocol. Nevertheless, its inclusion would strengthen the protocol's completeness and should be addressed before final acceptance.

Recommendation: Accept with minor revisions — request the authors to specify the estimated number of experts, selection criteria, and consultation method for the expert consultation stage as a condition for final acceptance.
