# Supplementary material for: Strategic communication to strengthen nurses’ professional identity and drive cultural change in healthcare organizations: A JBI scoping review protocol
Source: PLoS One. 2026 Sep 29;21(9):e0359546. doi: 10.1371/journal.pone.0359546 (PMC13623099; doi:10.1371/journal.pone.0359546)
Supplement: Supplementary file 14 [file pone.0359546.s012.pdf]

**EVALUATION REPORT: MANUSCRIPT REVISION COMPLIANCE ASSESSMENT**

**Evaluation Summary**

I have completed a comprehensive analysis of the revised manuscript (PONE-D-25-53788_R1) against the recommendations provided in the Consolidated Analysis Report. Below is a detailed assessment of each recommendation with compliance status and observations.

**Detailed Compliance Assessment**

| RECOMMENDATION | STATUS | OBSERVATIONS |
|---|---|---|
| **1. Results Presentation - Conceptual Clarity** Improve the presentation of results and conceptual clarity of the three theoretical frameworks (strategic communication, nurses' professional identity, cultural transformation) | **COMPLIANT** | The revised manuscript presents clearer definitions of the three theoretical frameworks. Strategic communication is explicitly defined as "intentional, organization-level practices aligned with institutional goals." The ISPIN-IBIN model is described with its 4 domains (Values, Knowledge, Leadership, Comportment). Cultural transformation is presented as a multidimensional process with 8 specific dimensions. |
| **2. Expert Consultation - Formal Implementation** Include a formal expert consultation phase after data synthesis for validation of findings | **PARTIALLY COMPLIANT** | The manuscript mentions the importance of expert consultation and dissemination of findings to nursing leadership and health communication communities. However, there is no detailed description of HOW this consultation will be structured, WHEN it will occur, WHO the consulted experts will be, or WHAT the specific protocol for this phase will be. Should include: specific timeline, expert selection criteria, feedback collection methodology, and plan for incorporating results. |
| **3. Protocol Registration - Transparency** Ensure protocol registration on the Open Science Framework (OSF) with DOI | **COMPLIANT** | The revised manuscript confirms protocol registration on OSF with DOI. This is documented in the transparency and accountability section of the document, demonstrating compliance with open research standards. |
| **4. PRISMA-ScR Compliance - Explicit Statement** | **COMPLIANT** | The revised manuscript includes explicit references to PRISMA-ScR guidelines and |

| RECOMMENDATION | STATUS | OBSERVATIONS |
|---|---|---|
| Include an explicit statement of compliance with PRISMA-ScR guidelines and reference the corresponding checklist | | the JBI (Joanna Briggs Institute) method. Compliance with these guidelines is clearly indicated in the methodology and protocol structure. |
| **5. Timeline - Detailed Project Schedule**<br>Incorporate a detailed project timeline with specific phases (Preparation, Search and Selection, Extraction and Analysis, Dissemination) with estimated durations and responsible parties | **NOT COMPLIANT** | The revised manuscript does NOT include a detailed timeline with the specific phases suggested. There is no information about: (a) estimated duration of each phase; (b) suggested periods for execution; (c) responsible parties for each step; (d) project milestones. The document mentions that the search covers records from 2000 to 2025, but does not present a temporal plan for the study's own execution. Should include a table or dedicated section with this information. |
| **6. Funding - Complete Transparency**<br>Maintain transparency regarding funding sources and ensure all sources are adequately disclosed | **COMPLIANT** | The revised manuscript maintains the statement that "the authors received no specific funding for this work." This is clearly documented in the Additional Information section, demonstrating complete transparency regarding funding status. |
| **7. Conflicts of Interest - Complete Declaration**<br>Ensure that the conflicts of interest declaration is complete and that all authors have declared appropriately | **COMPLIANT** | The revised manuscript includes the statement: "The authors have declared that no competing interests exist." All authors are listed with their institutional affiliations, and the conflicts of interest declaration is clear and comprehensive. |
| **8. Appendices - Detailed Search Strategy**<br>Include appendices with detailed search strategy for each database (PubMed, CINAHL, Web of Science, Scopus, etc.) | **PARTIALLY COMPLIANT** | The revised manuscript mentions that searches will be conducted in seven databases (PubMed, CINAHL, Web of Science, Scopus, and others). There is a "Search Strategy" section describing the general approach. However, it is unclear whether appendices with SPECIFIC strategies for each database are included in the document. Should include: exact |

| RECOMMENDATION | STATUS | OBSERVATIONS |
|---|---|---|
| | | search strings for each database, Boolean operators used, filters applied, and expected number of results per database. |
| **9. Appendices - Data Extraction Instrument** Include appendices with the detailed data extraction instrument to be used in the review | **PARTIALLY COMPLIANT** | The revised manuscript mentions that a data extraction instrument will be used, but does not present the complete or detailed instrument in the document body. It is unclear whether the instrument is included as an appendix. Should include: extraction form with all fields, operational definitions for each field, completion instructions, and examples of how to apply the instrument. |

**COMPLIANCE INDEX SUMMARY**

**Calculation:**

- **Fully Compliant:** 5 recommendations (1, 3, 4, 6, 7)
- **Partially Compliant:** 3 recommendations (2, 8, 9)
- **Non-Compliant:** 1 recommendation (5)
- **Total Recommendations:** 9

**Compliance Percentages:**

- **Fully Compliant:** 55.6% (5/9)
- **Partially Compliant:** 33.3% (3/9)
- **Non-Compliant:** 11.1% (1/9)

**Overall Compliance Index: 88.9%** (considering fully compliant + partially compliant)

**DETAILED ANALYSIS BY CATEGORY**

✓ **Fully Compliant Recommendations (5)**

1. **Conceptual Clarity** – Authors have significantly improved the presentation of the three theoretical frameworks with explicit and operational definitions.
2. **Protocol Registration** – Confirmed registration on OSF with DOI, meeting open research standards.
3. **PRISMA-ScR Compliance** – Explicit references to PRISMA-ScR guidelines and JBI methodology included.
4. **Financial Transparency** – Clear statement maintained regarding absence of specific funding.

5. **Conflicts of Interest Declaration** – Complete declaration of absence of conflicts of interest for all authors included.

⚠ **Partially Compliant Recommendations (3)**

**Recommendation 2 – Expert Consultation:**

- **Missing:** Detailed protocol with timeline, selection criteria, feedback methodology, and results incorporation plan.

**Recommendation 8 – Search Strategy:**

- **Missing:** Specific search strings for each database, Boolean operators, filters, and expected results per database.

**Recommendation 9 – Data Extraction Instrument:**

- **Missing:** Complete form with fields, operational definitions, completion instructions, and application examples.

❌ **Non-Compliant Recommendations (1)**

**Recommendation 5 – Timeline/Detailed Project Schedule:**

- **Status:** Completely absent from the revised manuscript.
- **Impact:** Critical for assessing project feasibility and planning.
- **Required:** Include table with phases (Preparation, Search and Selection, Extraction and Analysis, Dissemination), estimated durations, suggested periods, and responsible parties.

**FINAL ASSESSMENT**

The revised manuscript demonstrates **good progress** in addressing the recommendations, with **55.6% full compliance** and **88.9% overall compliance** (including partially compliant items).

**Strengths:** Conceptual clarity, transparency, and compliance with international guidelines.

**Critical areas requiring additional revision:**

1. **Detailed project timeline** (non-compliant)
2. **Specific expert consultation protocol** (partially compliant)
3. **Technical appendices** (search strategy and data extraction instrument – partially compliant)

**Recommendation:** Request that authors prioritize addressing the non-compliant recommendation (Timeline) and detail the three partially compliant recommendations before final acceptance.
