# Supplementary material for: Strategic communication to strengthen nurses’ professional identity and drive cultural change in healthcare organizations: A JBI scoping review protocol
Source: PLoS One. 2026 Sep 29;21(9):e0359546. doi: 10.1371/journal.pone.0359546 (PMC13623099; doi:10.1371/journal.pone.0359546)
Supplement: Supplementary file 15 [file pone.0359546.s014.pdf]

REVIEW REPORT — MANUSCRIPT PONE-D-25-53788_R2

Scoping Review Protocol: ***Strategic communication to strengthen nurses' professional identity and drive cultural change in healthcare organizations: A JBI Scoping Review Protocol***

Authors: Carmen Arteaga-Jordá, María Jesús Yáñez-Galdames, Hildegart González-Luis, Cristina Oroviogoicoechea, María José Hernández-Leal

Journal: PLOS One

PART 1 — CROSS-CUTTING ASSESSMENTS

1.1 Overall Methodological Quality

Assessment: Adequate ✅

The protocol rigorously follows the JBI methodology for scoping reviews, featuring:

- Research question structured in the PCC (Participants, Concept, Context) format
- Three-step search strategy per JBI recommendations
- Selection and extraction process with two independent reviewers
- Narrative synthesis with thematic organization
- Protocol registration on the Open Science Framework (DOI: 10.17605/OSF.IO/8KX7S)
- Declared compliance with PRISMA-ScR guidelines

1.2 Methodological Consistency

Assessment: Adequate ✅

Strong coherence exists across all stages of the protocol:

**Dimension**	**Coherence**
Objectives ↔ Methods	✅ Scoping review is the appropriate design for mapping the extent of the literature
PCC Question ↔ Eligibility Criteria	✅ Criteria derive directly from the PCC elements
PCC Question ↔ Data Extraction	✅ Extraction fields reflect the PCC elements
PCC Question ↔ Synthesis	✅ Thematic organization aligned with the three axes of the research question

Dimension	Coherence
Theoretical Frameworks ↔ Analysis	✓ The 14-component framework, ISPIN-IBIN model, and 8 dimensions anchor the analysis
Method ↔ Reporting Guidelines	✓ PRISMA-ScR is the appropriate guideline
Method ↔ Registration	✓ OSF is an appropriate platform for scoping review registration

No contradictions or methodological misalignments were identified.

1.3 Transparency and Reproducibility

Assessment: Adequate ✓

The review process is described with substantial detail that would allow an independent researcher to reproduce the review:

Element	Present?
Clear research question (PCC)	✓
Detailed eligibility criteria	✓
Specified databases (7 databases)	✓
Grey literature included	✓
Three-step search strategy (JBI)	✓
Selection process (2 independent reviewers)	✓
Data extraction (2 independent reviewers)	✓
Explicit theoretical frameworks	✓
PRISMA-ScR tool declared	✓
Protocol registration (OSF with DOI)	✓

Element	Present?
Full search strategy as appendix	⚠ Not confirmed
Data extraction instrument as appendix	⚠ Not confirmed

1.4 Management of Bias

Assessment: Requires revision ⚠

Strengths:

- The manuscript describes processes that indirectly mitigate bias: two independent reviewers for screening and extraction, a third reviewer for conflict resolution, multi-database search with no language restrictions, and inclusion of grey literature

Weaknesses:

- There is **no explicit discussion** of how potential biases may affect the results
- No section or paragraph dedicated to methodological limitations or biases
- The following are not addressed: publication bias, database selection bias (predominance of English-language literature regardless of no language restriction), inherent subjectivity of narrative synthesis, and limitations arising from the absence of critical quality appraisal of included studies

Suggestions for improvement:

- Add a brief discussion (2–3 paragraphs) addressing:
    1. **Inherent methodological limitations** — scoping reviews do not assess the methodological quality of included studies
    2. **Publication bias** — possible overrepresentation of contexts with greater academic output (USA, Europe, Australia)
    3. **Language bias** — despite no language restriction, databases predominantly index English-language literature
    4. **Mitigations adopted** — two independent reviewers, multiple databases, grey literature, expert consultation

1.5 Field Mapping

Assessment: Adequate ✅

The review proposes an effective mapping of the field, fulfilling the central purpose of a scoping review:

- Explicit objective to map the extent, range, and nature of the literature
- Analysis organized around the three theoretical frameworks, enabling identification of dense areas and gaps

- Declared intent to identify gaps in the literature and propose directions for future research
- Expert consultation as a complementary stage to strengthen gap identification

---

PART 2 — SECTION-BY-SECTION ASSESSMENTS

2.1 Title and Abstract

Assessment: Adequate ✅

Item	Assessment
Informative title	✅ Yes — "Strategic communication to strengthen nurses' professional identity and drive cultural change in healthcare organizations: A JBI Scoping Review Protocol"
Identified as scoping review/protocol	✅ Yes — explicit in the title
Structured abstract	✅ Yes — includes background, objectives, methods, and conclusions

2.2 Introduction and Rationale

Assessment: Adequate ✅

Strengths:

- Clear justification for the review, anchored in three gaps identified in the literature
- Robust contextualization of the three core concepts: strategic communication, nursing professional identity, and cultural transformation
- Three well-defined theoretical frameworks integrated into the rationale

2.3 Research Question (PCC)

Assessment: Adequate ✅

Strengths:

- Primary question clearly formulated in the PCC format
- Three specific sub-questions that unpack the main question
- Direct alignment between the PCC question and the eligibility criteria

Details:

- **Population (P):** Nurses in healthcare organizations
- **Concept (C):** Strategic communication

- **Context (C):** Cultural transformation in healthcare organizations

2.4 Eligibility Criteria

Assessment: Adequate ✅

Strengths:

- Inclusion and exclusion criteria clearly defined for each PCC element
- Table 1 (PCC Inclusion and Exclusion Summary) is well-structured
- Defined coverage period (2000–2025)
- No language restrictions
- Inclusion of diverse source types (qualitative, quantitative, mixed methods, theses, grey literature)

2.5 Information Sources and Search Strategy

Assessment: Adequate ✅

Strengths:

- Seven bibliographic databases specified: PubMed, CINAHL, Scopus, Web of Science, PsycINFO, Cochrane Library, LILACS
- Grey literature included: Google Scholar, OpenGrey, institutional repositories, WHO, ICN
- Three-step search strategy per JBI methodology
- Adaptation of the strategy for each database mentioned
- Defined period (2000–2025) with no language restrictions

Weaknesses:

- The **full search strategy** for at least one primary database (e.g., PubMed with MeSH terms, Boolean operators, free-text keywords, and filters) **was not confirmed as included as an appendix** in the material analyzed
- The referenced S1 Appendix pertains to the *Strategic communication framework*, not the search strategy

Suggestions for improvement:

- Include as an appendix the complete search strategy for MEDLINE/PubMed, containing: database name, search date, full string (MeSH terms + keywords + Boolean operators), applied filters, and number of results obtained

2.6 Study Selection Process (Screening)

Assessment: Adequate ✅

Strengths:

- Two clear stages: (1) title and abstract screening, (2) full-text reading

- **Two independent reviewers** at both stages (peer review)
- Explicit conflict resolution mechanism: consensus or third reviewer
- PRISMA-ScR flowchart for documenting the process
- Recording of exclusion reasons at the full-text stage

2.7 Data Extraction

Assessment: Adequate ✅ (with caveat)

Strengths:

- Two independent reviewers for extraction
- Conflict resolution by consensus or third reviewer
- Standardized form based on the JBI model
- Extraction fields listed: author(s), year, country, objective, population, concept, context, study design, key findings

Weaknesses:

- The **complete extraction instrument** (template or form) **was not confirmed as included as an appendix** in the material analyzed

Suggestions for improvement:

- Include as an appendix the complete data extraction instrument, in table or checklist format, containing all fields to be extracted from each study

2.8 Data Synthesis and Presentation

Assessment: Adequate ✅

Strengths:

- Narrative and descriptive synthesis — the standard and appropriate approach for scoping reviews
- Data organized into thematic categories aligned with the three axes of the review
- Presentation in tables (summarizing study characteristics) and figures (conceptual mapping)
- Analysis anchored in the three theoretical frameworks:
    - 14 components of strategic communication
    - ISPIN-IBIN model of professional identity
    - 8 dimensions of cultural transformation
- Identification of gaps and directions for future research
- PRISMA-ScR flowchart for documenting the selection process

2.9 Expert Consultation

Assessment: Adequate ✅

Strengths:

- Additional stage included, following JBI methodology
- Experts in strategic communication, nursing professional identity, and organizational change will be consulted
- Feedback will be used to validate and enrich the interpretation of findings
- Appropriate positioning: after initial data analysis

2.10 Timeline

Assessment: Adequate ✅

Stage	Timeline
Database search	Months 1–2
Study selection (screening)	Months 2–3
Data extraction	Months 3–4
Data analysis and synthesis	Months 4–5
Expert consultation	Month 5
Manuscript writing	Months 5–6
Submission	Month 6

Observation: The total 6-month timeline is realistic for a scoping review conducted by an experienced team.

2.11 Currency of the Search

Assessment: Adequate ✅

- Search scheduled for months 1–2 of the 6-month timeline
- Broad coverage period (2000–2025)
- Provision for an updated search before publication if a significant delay occurs between the search and submission

2.12 Ethics and Dissemination

Assessment: Adequate ✅

- Ethics approval waiver declared (scoping review of published literature)
- Dissemination plan: publication in a peer-reviewed journal, conference presentations, communication to stakeholders

2.13 Funding

Assessment: Adequate ✅

- Funding source declared: CAPES (Coordenação de Aperfeiçoamento de Pessoal de Nível Superior) — Brazil
- Funding code: 001
- Explicit declaration of no funder involvement in the design, conduct, or reporting of the review

2.14 Conflicts of Interest

Assessment: Adequate ✅

- Specific section included (*Competing Interests*)
- Explicit negative declaration: authors declare no competing interests
- Declaration that no funding was received from commercial or non-academic agencies

2.15 Author Contributions

Assessment: Adequate ✅

- Contributions described in both narrative and CRediT format
- Individual roles clearly attributed

2.16 References

Assessment: Adequate ✅

- Numbered reference list included
- Format compatible with journal standards

2.17 Appendices

Assessment: Requires revision ⚠

Appendices identified in the manuscript:

- **S1 Appendix:** Strategic communication framework
- **S2 Appendix:** ISPIN-IBIN nursing professional identity model
- **S3 Appendix:** Eight-dimension cultural transformation framework
- **S4 Appendix:** Search strategy (mentioned, but content not confirmed)
- **S5 Appendix:** Data extraction instrument (mentioned, but content not confirmed)

Weaknesses:

- The **full search strategy** (S4 Appendix) **was not confirmed as included** in the material analyzed
- The **data extraction instrument** (S5 Appendix) **was not confirmed as included** in the material analyzed

Suggestions for improvement:

- Verify that S4 Appendix and S5 Appendix are effectively included in the submission package
- If not included, add them as supplementary material to ensure transparency and reproducibility

---

PART 3 — SUMMARY TABLE OF ASSESSMENTS

#	Item	Assessment
1	Title and Abstract	✅ Adequate
2	Introduction and Rationale	✅ Adequate
3	Research Question (PCC)	✅ Adequate
4	Eligibility Criteria	✅ Adequate
5	Information Sources	✅ Adequate
6	Search Strategy	✅ Adequate
7	Full Search Strategy (Appendix)	⚠ Requires revision
8	Study Selection Process (Screening)	✅ Adequate
9	Data Extraction — Process	✅ Adequate
10	Data Extraction Instrument (Appendix)	⚠ Requires revision
11	Data Synthesis and Presentation	✅ Adequate
12	Expert Consultation	✅ Adequate

#	Item	Assessment
13	Protocol Registration	✅ Adequate
14	PRISMA-ScR Compliance	✅ Adequate
15	Timeline	✅ Adequate
16	Currency of the Search	✅ Adequate
17	Funding	✅ Adequate
18	Conflicts of Interest	✅ Adequate
19	Author Contributions	✅ Adequate
20	References	✅ Adequate
21	Transparency and Reproducibility	✅ Adequate
22	Management of Bias	⚠ Requires revision
23	Methodological Consistency	✅ Adequate
24	Field Mapping	✅ Adequate

Total: 21 ✅ Adequate | 3 ⚠ Requires revision

PART 4 — PRIORITY RECOMMENDATIONS

Mandatory Recommendations (to fully meet PRISMA-ScR and JBI standards)

1. **Full search strategy as appendix** — Include the complete search string for MEDLINE/PubMed (or another primary database) with MeSH terms, Boolean operators, free-text keywords, filters, and number of results. S4 Appendix should be verified and, if absent, added.
2. **Data extraction instrument as appendix** — Make the complete extraction form/template available as supplementary material (S5 Appendix), containing all fields to be extracted from each study.

3. **Discussion of limitations and biases** — Add a section or paragraph acknowledging the inherent limitations of the scoping review method (absence of critical quality appraisal, publication bias, language bias, subjectivity of narrative synthesis) and the mitigations adopted.

Secondary Recommendations (would strengthen the protocol)

4. **Verify the effective inclusion** of all mentioned appendices (S1–S5) in the submission package.
5. **Detail the expert consultation plan** — Specify the estimated number of experts, selection criteria, and consultation method (e.g., Delphi, interviews, focus group).

PART 5 — STRENGTHS OF THE MANUSCRIPT

1. **Robust methodology** — Rigorously follows JBI and PRISMA-ScR guidelines, incorporating all essential elements of a scoping review protocol.
2. **Well-integrated theoretical frameworks** — Three complementary models (strategic communication, ISPIN-IBIN, cultural transformation) anchor the analysis coherently.
3. **Peer-reviewed selection process** — Two independent reviewers at all stages (screening and extraction), with a clear conflict resolution mechanism.
4. **Comprehensive search** — Seven databases, grey literature, no language restrictions, 25-year coverage period.
5. **Protocol registration** — OSF with DOI available for verification.
6. **Expert consultation** — Additional stage that strengthens the validity and practical relevance of the findings.
7. **Internal consistency** — Strong coherence among objectives, methods, and expected results.
8. **Financial transparency** — Funding declared with explicit statement of non-interference.

PART 6 — FINAL CONSIDERATIONS

Manuscript PONE-D-25-53788_R2 presents a **high-quality scoping review protocol**, demonstrating methodological maturity in the application of JBI and PRISMA-ScR guidelines. As a revised submission (R2), it has already incorporated feedback from prior rounds of peer review, which is reflected in the robustness of the majority of assessed items.

The three areas requiring revision are **specific and readily correctable**: effective inclusion of the full search strategy and data extraction instrument as appendices, and the addition of a brief discussion of limitations and biases. None of these compromise the methodological validity of the protocol.

The study addresses a relevant and underexplored topic — the intersection of strategic communication, nursing professional identity, and cultural transformation in healthcare

organizations — with the potential for significant contributions to practice, policy, and research in the field.

Overall recommendation: Accept with minor revisions, contingent upon the inclusion of the search and extraction appendices, and the addition of a discussion of limitations and biases.
