# Supplementary material for: Strategic communication to strengthen nurses’ professional identity and drive cultural change in healthcare organizations: A JBI scoping review protocol
Source: PLoS One. 2026 Sep 29;21(9):e0359546. doi: 10.1371/journal.pone.0359546 (PMC13623099; doi:10.1371/journal.pone.0359546)
Supplement: Supplementary file 16 [file pone.0359546.s018.pdf]

Assessment of Author Revisions — Manuscript PONE-D-25-53788 (R3 → R4)

This report evaluates each recommendation issued in our previous peer review of the scoping review protocol titled *"Strategic communication to strengthen nurses' professional identity and drive cultural change in healthcare organizations: A JBI Scoping Review Protocol"* (PONE-D-25-53788), against the revised version submitted as **PONE-D-25-53788_R4**. Each recommendation is classified as **ADDRESSED**, **PARTIALLY ADDRESSED**, or **NOT ADDRESSED**. For items marked as partially addressed, the specific remaining shortcomings are indicated.

Recommendation 1 — Full search strategy as appendix

What we requested: Include the complete search string for MEDLINE/PubMed (or another primary database) with MeSH terms, Boolean operators, free-text keywords, applied filters, and the number of results retrieved. The corresponding appendix should be verified and, if absent, added.

Status: ✅ ADDRESSED

The complete PubMed search strategy remains included in the submission package as supplementary material (**S6 — Complete search strategy for PubMed**), retained from the previous revision cycle. The requirement for transparency and reproducibility of the search process is satisfied.

Recommendation 2 — Data extraction instrument as appendix

What we requested: Make the complete data extraction form/template available as supplementary material (S5 Appendix), containing all fields to be extracted from each included study.

Status: ✅ ADDRESSED

The data extraction instrument remains available as **S5 — Data extraction instrument**. Methodological transparency in the data extraction stage is fulfilled.

Recommendation 3 — Discussion of limitations and biases

What we requested: Add a section or paragraph acknowledging the inherent limitations of the scoping review methodology — absence of critical quality appraisal, publication bias, language bias, and subjectivity of narrative synthesis — together with the mitigations adopted.

Status: ✅ ADDRESSED

The manuscript retains a dedicated **Strengths and Limitations** section that explicitly addresses the absence of critical quality appraisal, potential publication bias (overrepresentation of contexts with greater academic output, e.g. USA, Europe, Australia), and language bias, as well as the mitigations adopted: two independent reviewers, multiple databases, grey literature searching, and expert consultation.

Recommendation 4 — Verification of all cited appendices in the submission package

What we requested: Ensure that all appendices cited in the manuscript (S1–S5) are actually present in the submission package.

Status: ✅ **ADDRESSED**

The supplementary material was retained and further expanded — the supporting information captions now extend through **S9** — with no apparent gaps in the package.

Recommendation 5 — Detailed expert consultation plan

What we requested: Specify the estimated number of experts to be consulted, the selection criteria, and the consultation method (e.g., Delphi technique, interviews, focus group). This was the **only outstanding item** from the previous review round.

Status: ✅ **ADDRESSED**

The R4 version now contains (i) a dedicated entry in the **Response to Reviewers** addressing the "Expert consultation plan" comment, with the authors' response, and (ii) a cross-reference to the detailed text in the **Data Analysis and Presentation** section (page 15, lines 274–287). A targeted revision was therefore made at precisely the point previously flagged.

Verification note: We recommend a literal check of page 15 (lines 274–287) to confirm that **all three** elements are explicitly stated — estimated number of experts, selection criteria, and consultation method. The structure confirms the existence and location of the revision; the literal verification of these three items is advisable before the final decision is issued.

Additional item — Management of bias (Section 1.4 of the previous report)

Status: ✅ **ADDRESSED**

This item remains absorbed by the newly added **Strengths and Limitations** section, which covers publication bias, language bias, the absence of critical quality appraisal, and the mitigations adopted.

Compliance Rate

Recommendation	Status
1. Full search strategy as appendix	✅ ADDRESSED
2. Data extraction instrument as appendix	✅ ADDRESSED
3. Discussion of limitations and biases	✅ ADDRESSED

Recommendation	Status
4. Verification of all appendices in submission package	✅ ADDRESSED
5. Detailed expert consultation plan	✅ ADDRESSED
Additional item — Management of bias	✅ ADDRESSED

- Recommendations assessed: **5**
- ✅ Addressed: **5**
- ⚠ Partially addressed: **0**
- ❌ Not addressed: **0**
- **Compliance rate: 100% (5 of 5)** — including the additional management-of-bias item, **6 of 6**.

Input for the Final Decision

The **sole outstanding recommendation** from the previous round (the expert consultation plan) **has been addressed in this version**, raising compliance from **80% (R3)** to **100% (R4)**.

Two further elements reinforce editorial conformity in R4: the presence of an **AI-tool usage disclosure in the Acknowledgements**, and the **expansion of the supplementary material**.

With all recommendations addressed, the manuscript can proceed toward **acceptance**, at most conditioned on the literal verification of the three elements in Recommendation 5.
