# Supplementary material for: Strategic communication to strengthen nurses’ professional identity and drive cultural change in healthcare organizations: A JBI scoping review protocol
Source: PLoS One. 2026 Sep 29;21(9):e0359546. doi: 10.1371/journal.pone.0359546 (PMC13623099; doi:10.1371/journal.pone.0359546)
Supplement: Supplementary file 17 [file pone.0359546.s013.docx]

**Hildegart González-Luis, PhD**
University of Navarra

15th of May 2026

**Udoka Okpalauwaekwe, MD, MPH, PhD**

Academic Editor

PLOS One

**RE: Resubmission of PONE-D-25-53788**

Dear Dr. Okpalauwaekwe,

We are pleased to resubmit our manuscript entitled:

**“The role of strategic communication in integrating cultural change and reinforcing nurses’ professional identity in healthcare organizations: a scoping review protocol”** (PONE-D-25-53788) for consideration in *PLOS ONE*.

We sincerely thank you and Reviewer #1 (Emerson Roberto dos Santos, MSc) for the constructive and insightful feedback. The revisions have significantly enhanced the manuscript's conceptual clarity, methodological transparency, and analytical depth, while addressing all points raised.

In accordance with your recommendations, we have:

- Clarified the interaction between the three core concepts—strategic communication, cultural transformation, and nurses’ professional identity—and their distinct roles in inclusion criteria, data extraction, and analysis, with explicit management of framework overlaps.
- Refined the operational definition of strategic communication as intentional, organization-level practices aligned with institutional goals to influence professional identity and organizational culture, ensuring consistency during screening.
- Strengthened the analytical contribution by incorporating an explicit interpretative layer within the convergent synthesis, enabling the exploration of relationships beyond descriptive mapping.
- Clarified the handling of opinion pieces and editorials: these will be included only when providing substantive conceptual contributions, analysed separately from empirical studies, and clearly identified in the synthesis (none are currently expected based on preliminary screening).
- Incorporated an optional post-synthesis expert consultation step to enhance interpretative rigor and practical relevance.
- Added a tentative timeline for the review phases as Supplementary Information (S5 Appendix).

These revisions are minor in nature, do not alter the core study design or methodology, and are fully detailed in the accompanying point-by-point Response to Reviewers document and visible throughout the tracked-changes manuscript.

**Submitted Files:**

- Clean version (Manuscript)

- Tracked-changes version of the manuscript

- Point-by-point Response to Reviewers

**Data Availability Statement:** Data sharing is not applicable to this article as no datasets were generated or analyzed during the current scoping review protocol.

We confirm that this manuscript remains original, unpublished, and approved by all authors.

Thank you again for your time and continued consideration of our work. We look forward to your response.

Sincerely,

Hildegart González-Luis, PhD
On behalf of all co-authors

# Point-by-point Response to Reviewer #1 (Emerson Roberto dos Santos, MsS)

| **Response to Review Decision #1** | **Authors Answers** |
| --- | --- |
| *“Additional Editor Comments:”* | We thank the Reviewer, Emerson Roberto dos Santos, for his careful evaluation and constructive feedback. We have addressed all comments and revised the manuscript accordingly. All changes have been incorporated into the revised manuscript and are indicated with tracked changes. |
| Comment 1: Conceptual complexity and use of multiple frameworks  *“Well written protocol but conceptually over-engineered. The simultaneous use of multiple frameworks (14-component communication model, ISPIN-IBIN, and cultural transformation framework) risks adding complexity without clear justification of how each uniquely contributes to the analysis.*  *Relatedly, it is unclear whether these frameworks are guiding inclusion, analysis, or both, and how overlap between them will be managed.”* | We thank the reviewer for this insightful comment. We agree that clarifying both the interaction between the three core concepts and the specific role of each framework is essential to ensure conceptual coherence and methodological transparency.  In response, we have revised the *Concepts* section to explicitly prioritise the inclusion of studies that demonstrate an explicit interaction between strategic communication, cultural transformation, and nurses’ professional identity, rather than their isolated presence. Specifically, eligible studies must reflect a relationship in which strategic communication operates as an intentional organizational mechanism within processes of institutional cultural transformation, influencing the construction or reinforcement of professional identity.  We have also clarified the distinct methodological role of each framework. While the inclusion criteria are based on the interaction between the three core concepts, the frameworks are primarily used to guide data extraction and analysis. In this regard, the strategic communication framework supports the identification and categorisation of communication practices; the ISPIN-IBIN framework informs the interpretation of professional identity dimensions; and the cultural transformation framework provides the organizational context for analysis.  To ensure consistency between the narrative description and the operational criteria, we have also revised Table 1 to reflect this interaction-based inclusion approach, and clarified participant eligibility criteria, and explicitly excluded studies where the three core concepts are not meaningfully and explicitly connected. The abstract has been updated accordingly.  These revisions are intended to reduce conceptual ambiguity, clarify the analytical contribution of each framework, and strengthen the overall coherence of the study design (see clean manuscript, *Abstract* section, page 2, lines 31, 35-38; *Concepts* section, page 7, lines 142–146,158-160, 182-188; page 9-10, Table 1). |
| Comment 2: Definition of strategic communication  *“The definition of “strategic communication” is broad and somewhat circular, which may introduce inconsistency in study selection.”* | We thank the reviewer for highlighting the need to refine the definition of strategic communication. We agree that a more operational and explicit definition is essential to ensure consistency in study selection.  In response, we have revised the *Concepts* section to provide a clearer and more focused operational definition. Strategic communication is now defined as intentional, organization-level communication practices aligned with institutional goals and designed to influence professional identity and organizational culture.  This definition is explicitly linked to the inclusion criteria, ensuring that only studies addressing communication as an organizational and purposeful mechanism—rather than routine, technical, or interpersonal communication—are considered.  These revisions aim to reduce conceptual ambiguity and improve consistency during the screening and selection process. This clarification also aligns with the interaction-based inclusion approach described in the revised eligibility criteria (see clean manuscript, *Concepts* section, page 7, lines 151–153). |
| Comment 3: Inclusion of editorials and opinion pieces  *“Inclusion of opinion pieces and editorials raises concerns about conceptual dilution unless clearly distinguished from empirical evidence in synthesis.”* | We thank the reviewer for this important observation. We agree that the inclusion of opinion and editorial sources requires careful handling to avoid conceptual dilution.  In response, we have revised the *Types of sources* section to clarify that such sources will only be included when they provide substantive conceptual contributions relevant to the review objectives. Furthermore, they will be analysed separately from empirical studies and clearly identified in the synthesis.  This approach ensures transparency and preserves the distinction between empirical evidence and conceptual contributions. We also note that, based on our preliminary screening, no opinion or editorial sources are currently expected to be included; however, this clarification ensures methodological consistency should such sources be identified during the review process (see clean manuscript, *Types of sources* section, page 12, lines 211–212). |
| Comment 4: Mapping vs. interpretative synthesis  *“While methodologically sound, the protocol leans heavily toward mapping rather than critical synthesis, which may limit its contribution.”* | We thank the reviewer for this important observation. We agree that clarifying the analytical contribution of the review is essential.  In response, we have revised the *Data analysis and presentation* section to explicitly incorporate an interpretative layer alongside the descriptive mapping approach. Specifically, beyond summarizing and mapping the evidence, the review will explore relationships between communication practices, professional identity construction, and cultural transformation processes. The abstract has also been updated accordingly.  This interpretative component is consistent with our use of a convergent synthesis approach and the application of three complementary analytical frameworks, which together enable a more in-depth examination of patterns, relationships, and contextual dynamics across studies.  These revisions aim to enhance the analytical depth of the review while remaining consistent with the methodological purpose of a scoping review (see clean manuscript, *Abstract* section, page 2, lines 42-44; *Data analysis and presentation* section, page 14-15, lines 267–274). |
| Comment 5. Include a Formal Post-Synthesis Consultation Step.  *“It is recommended to consider including a formal expert or stakeholder consultation step after the preliminary synthesis of results. This step could involve presenting the findings to a panel of experts (nurses, healthcare leaders, communication specialists) to gather feedback on data interpretation, identify additional gaps, and validate the relevance and practicality of the implications and recommendations. This would further align the protocol with best practices for scoping reviews and enhance the impact of the results.”* | We thank the reviewer for this valuable suggestion. We agree that incorporating an expert consultation stage can enhance the interpretative robustness and practical relevance of the review.  In response, we have revised the *Methods* section (*Data analysis and presentation*) to include an optional post-synthesis consultation step. We also highlight the multidisciplinary composition of the research team—encompassing expertise in strategic communication, clinical leadership in cultural transformation processes associated with Magnet accreditation, and doctoral-level research training—as a key strength supporting the analytical process.  To further strengthen analytical rigor, we have incorporated an optional external consultation following preliminary synthesis, engaging an independent international expert to contrast interpretations and enhance conceptual coherence. This consultation will be conducted as a reflexive, non-structured process aimed at enriching the discussion rather than systematically modifying the results.  These revisions aim to enhance the interpretative depth and practical relevance of the review while maintaining methodological flexibility consistent with scoping review guidance (see clean manuscript, *Data analysis and presentation* section, page 15, lines 274–277). |
| Comment 6. Add a Detailed Timeline.  *“It is essential to add a detailed tentative timeline specifying the estimated durations for each phase of the review. This not only aids in project planning and execution but also enhances the transparency of the protocol. Below, I provide a timeline template that could be adapted and included.”* | We thank the reviewer for this valuable suggestion. We agree that including a timeline enhances transparency and facilitates planning and reproducibility of the review process.  In response, we have incorporated a statement in the Methods section indicating that a tentative timeline—adapted from the reviewer template—outlining the main phases of the scoping review (search, screening, data extraction, analysis, and dissemination) is provided as Supplementary Information (S5 Appendix). Accordingly, the numbering of subsequent appendices has been updated for consistency throughout the manuscript.  This addition aims to improve the clarity of the review process while maintaining flexibility, in line with the iterative nature of scoping review methodology (see clean manuscript, *Methods* section, page 12, lines 222–223). |
|  | These minor revisions strengthen the protocol without altering its design. |
