# Supplementary material for: Strategic communication to strengthen nurses’ professional identity and drive cultural change in healthcare organizations: A JBI scoping review protocol
Source: PLoS One. 2026 Sep 29;21(9):e0359546. doi: 10.1371/journal.pone.0359546 (PMC13623099; doi:10.1371/journal.pone.0359546)
Supplement: Supplementary file 18 [file pone.0359546.s015.docx]

**Response to Reviewer Comments**

We thank the reviewer for the positive reassessment of the revised manuscript and for the constructive final recommendations. In response to the remaining points identified as non-compliant or partially compliant, we have made the revisions detailed below. The table summarizes the actions taken and the location of the corresponding changes in the revised submission.

**Detailed Compliance Assessment**

| **RECOMMENDATION** | **REVIEWER REASSESSMENT** | **AUTHORS ANSWERS** |
| --- | --- | --- |
| **2. Expert Consultation - Formal Implementation**  Include a formal expert consultation phase after data synthesis for validation of findings | **PARTIALLY COMPLIANT**. The manuscript mentions the importance of expert consultation and dissemination of findings to nursing leadership and health communication communities. However, there is no detailed description of HOW this consultation will be structured, WHEN it will occur, WHO the consulted experts will be, or WHAT the specific protocol for this phase will be. Should include: specific timeline, expert selection criteria, feedback collection methodology, and plan for incorporating results. | To address this recommendation, we expanded the description of the expert consultation phase. The revised manuscript now specifies the timing of the consultation (following preliminary synthesis and prior to final interpretation of findings), expert selection criteria (one independent international expert with recognized expertise in nursing leadership, professional identity, strategic communication, or organizational transformation), feedback collection methodology (written comments and/or a virtual consultation), and the intended use of feedback. Feedback will be considered during refinement of the discussion and conceptual integration of findings but will not modify study eligibility criteria, extracted data, or primary findings.  These revisions provide greater transparency regarding the consultation process and its intended contribution to the interpretation of findings.  Location:  See revised manuscript, Data Analysis and Presentation section, page 15, lines 277–285). |
| **5. Timeline - Detailed Project Schedule**  Incorporate a detailed project timeline with specific phases (Preparation, Search and Selection, Extraction and Analysis, Dissemination) with estimated durations and responsible parties | **NOT COMPLIANT**. The revised manuscript does NOT include a detailed timeline with the specific phases suggested. There is no information about: (a) estimated duration of each phase; (b) suggested periods for execution; (c) responsible parties for each step; (d) project milestones. The document mentions that the search covers records from 2000 to 2025, but does not present a temporal plan for the study's own execution. Should include a table or dedicated section with this information. | We thank the reviewer for this observation A detailed tentative timeline was included as S5 Appendix in the Supplementary Information document and explicitly referenced in the Methods section. The appendix outlines the major phases of the review, estimated duration, suggested execution periods (months), and responsible contributors across the review process. As this information is operational in nature, it was provided in supplementary material and referenced within the Methods section.  Location:  Methods section, page 12, lines 221–223 and page 25, lines 561-562; S5 Appendix (Supplementary Information). |
| **8. Appendices - Detailed Search Strategy**  Include appendices with detailed search strategy for each database (PubMed, CINAHL, Web of Science, Scopus, etc.) | **PARTIALLY COMPLIANT**. The revised manuscript mentions that searches will be conducted in seven databases (PubMed, CINAHL, Web of Science, Scopus, and others). There is a "Search Strategy" section describing the general approach. However, it is unclear whether appendices with SPECIFIC strategies for each database are included in the document. Should include: exact search strings for each database, Boolean operators used, filters applied, and expected number of results per database. | We thank the reviewer for this observation. The search strategy presented in S6 Appendix was developed in accordance with JBI methodological guidance for scoping review protocols and includes a complete reproducible search strategy for MEDLINE (via PubMed), including search terms, Boolean operators, controlled vocabulary, search limits, search date, number of records retrieved, and PRESS validation documentation. To address the reviewer’s request for greater detail regarding database-specific implementation, we have additionally included S7 Appendix, which provides the adapted search strategies used across all databases searched, including database-specific syntax and operators where applicable. This supplementary material is intended to further enhance transparency and facilitate reproducibility of the search process.  Location: Search Strategy section, page 13, lines 237–238 and page 25, lines 559-560; S6 and S7 Appendix (Supplementary Information). |
| **9. Appendices - Data Extraction Instrument**  Include appendices with the detailed data extraction instrument to be used in the review | **PARTIALLY COMPLIANT**. The revised manuscript mentions that a data extraction instrument will be used, but does not present the complete or detailed instrument in the document body. It is unclear whether the instrument is included as an appendix. Should include: extraction form with all fields, operational definitions for each field, completion instructions, and examples of how to apply the instrument. | We thank the reviewer for this observation. The complete data extraction instrument is provided in S8 Appendix (Supplementary Information). The appendix includes all extraction fields, operational definitions, coding instructions, and an illustrative example demonstrating how the instrument may be applied and coded. The example is provided for demonstration purposes only and does not correspond to any study included in the review.  Data Extraction section, page 14, lines 255–256 and page 25, lines 561-563; S8 Appendix (Supplementary Information). |
