# Supplementary material for: Strategic communication to strengthen nurses’ professional identity and drive cultural change in healthcare organizations: A JBI scoping review protocol
Source: PLoS One. 2026 Sep 29;21(9):e0359546. doi: 10.1371/journal.pone.0359546 (PMC13623099; doi:10.1371/journal.pone.0359546)
Supplement: Supplementary file 19 [file pone.0359546.s017.docx]

**Response to Reviewer Comments**

We thank the editorial team for the careful assessment of our revised manuscript and for the constructive final comments. The table below provides a focused point-by-point response to the actionable recommendations listed in Part 4 — Priority Recommendations of the assessment report. To facilitate traceability while avoiding unnecessary repetition, the comments are reproduced using the wording of Part 4, followed by our response and the corresponding location in the revised manuscript or supplementary information.

**Response to Part 4 — Priority Recommendations**

| **Part 4 priority recommendation** | **Authors’ response** |
| --- | --- |
| **Mandatory recommendation 1 — Full search strategy as appendix**  Full search strategy as appendix — Include the complete search string for MEDLINE/PubMed (or another primary database) with MeSH terms, Boolean operators, free-text keywords, filters, and number of results. S4 Appendix should be verified and, if absent, added. | We appreciate this observation. We clarify that the full search strategy is provided in the final supplementary information as S6 Appendix, not S4 Appendix. The complete reproducible MEDLINE (via PubMed) search strategy is included in S6 Appendix, and the database-specific adaptations for the remaining information sources are included in S7 Appendix. We have verified the consistency of the appendix numbering and supplementary file labels throughout the manuscript and supplementary information.  **Location:** Manuscript, Search strategy section, page 13, lines 235–238; Supplementary Information, S6 Appendix and S7 Appendix. |
| **Mandatory recommendation 2 — Data extraction instrument as appendix**  Data extraction instrument as appendix — Make the complete extraction form/template available as supplementary material (S5 Appendix), containing all fields to be extracted from each study. | We appreciate this observation. We clarify that the data extraction instrument is provided in the final supplementary information as S8 Appendix, not S5 Appendix. The complete standardized and piloted data extraction instrument is included in S8 Appendix, including the extraction fields, operational definitions, coding guidance, and an illustrative example. We have verified the consistency of the appendix numbering and supplementary file labels throughout the manuscript and supplementary information.  **Location:** Manuscript, Data extraction section, page 14, line 255; Supplementary Information, S8 Appendix. |
| **Mandatory recommendation 3 — Discussion of limitations and biases**  Discussion of limitations and biases — Add a section or paragraph acknowledging the inherent limitations of the scoping review method (absence of critical quality appraisal, publication bias, language bias, subjectivity of narrative synthesis) and the mitigations adopted. | We appreciate this valuable suggestion. We expanded the Strengths and Limitations section to provide a more explicit discussion of methodological limitations and potential sources of bias relevant to this scoping review. The revised manuscript now acknowledges the absence of formal critical appraisal, publication bias, language bias, conceptual heterogeneity, inconsistent terminology or indexing, and the limitations associated with mapping and categorising communication strategies without assessing effectiveness or impact. In addition, we describe the methodological safeguards incorporated into the protocol, including a comprehensive peer-reviewed search strategy, independent dual screening and data extraction, calibration procedures, and predefined coding frameworks to enhance transparency, consistency, and reproducibility.  **Location:** Manuscript, Strengths and Limitations section, pages 18–19, lines 353–367. |
| **Secondary recommendation 4 — Verification of supplementary files**  Verify the effective inclusion of all mentioned appendices (S1–S5) in the submission package. | We appreciate this observation. We clarify that the final supplementary information includes nine supplementary files (S1–S9). The full search strategy is provided in S6 Appendix and the data extraction instrument is provided in S8 Appendix. We have verified the consistency of all appendix citations, appendix numbering, and supplementary file labels throughout the manuscript and supplementary information.  **Location:** Manuscript, Supporting information captions, page 26, lines 562–576; Supplementary Information, S1–S9 Appendices. |
