# Supplementary material for: Strategic communication to strengthen nurses’ professional identity and drive cultural change in healthcare organizations: A JBI scoping review protocol
Source: PLoS One. 2026 Sep 29;21(9):e0359546. doi: 10.1371/journal.pone.0359546 (PMC13623099; doi:10.1371/journal.pone.0359546)
Supplement: Supplementary file 20 [file pone.0359546.s019.docx]

**Response to Reviewer Comments**

We thank the reviewer for the reassessment of our manuscript. We have addressed the remaining recommendation identified for final acceptance as detailed below your check list review.

Final Assessment Manuscript PONE-D-25-53788_R3 has satisfactorily addressed all mandatory (priority) recommendations from the previous review round. The complete search strategy for PubMed has been included as a supplementary appendix, the data extraction instrument has been made available, and a dedicated discussion of methodological limitations and biases has been incorporated into the manuscript. These changes substantially enhance the transparency, reproducibility, and methodological rigor of the protocol.

The sole outstanding recommendation — detailing the expert consultation plan — is a secondary recommendation that does not compromise the methodological validity of the protocol. Nevertheless, its inclusion would strengthen the protocol's completeness and should be addressed before final acceptance. Recommendation: Accept with minor revisions — request the authors to specify the estimated number of experts, selection criteria, and consultation method for the expert consultation stage as a condition for final acceptance

| **Reviewer comment** | **Authors’ response** |
| --- | --- |
| **Detailed expert consultation plan**  Specify the estimated number of experts, selection criteria, and consultation method. | We appreciate this constructive suggestion. In response to the reviewer's recommendation, we have further elaborated the expert consultation stage. The revised manuscript now specifies that one independent international expert will be invited, outlines the expert selection criteria, and describes the consultation method (written comments and/or a virtual consultation). These additions provide greater clarity regarding the consultation process while preserving its intended role as an interpretative step to enhance analytical rigor rather than to modify the review findings.  **Location:** *Data Analysis and Presentation* section, page 15, lines 274–287. |
