# Supplementary material for: Strategic communication to strengthen nurses’ professional identity and drive cultural change in healthcare organizations: A JBI scoping review protocol
Source: PLoS One. 2026 Sep 29;21(9):e0359546. doi: 10.1371/journal.pone.0359546 (PMC13623099; doi:10.1371/journal.pone.0359546)
Supplement: Peer review history

# Peer review: Decision Letter 0

**DOI:** 10.1371/journal.pone.0359546.r001

**Authors:** Udoka Okpalauwaekwe

8 Apr 2026

Dear Dr. González Luis,

Please submit your revised manuscript by May 23 2026 11:59PM. If you will need more time than this to complete your revisions, please reply to this message or contact the journal office at plosone@plos.org. When you're ready to submit your revision, log on to https://www.editorialmanager.com/pone/ and select the 'Submissions Needing Revision' folder to locate your manuscript file.

- A letter that responds to each point raised by the academic editor and reviewer(s). You should upload this letter as a separate file labeled 'Response to Reviewers'.
- A marked-up copy of your manuscript that highlights changes made to the original version. You should upload this as a separate file labeled 'Revised Manuscript with Track Changes'.
- An unmarked version of your revised paper without tracked changes. You should upload this as a separate file labeled 'Manuscript'.

If you would like to make changes to your financial disclosure, please include your updated statement in your cover letter. Guidelines for resubmitting your figure files are available below the reviewer comments at the end of this letter.

If applicable, we recommend that you deposit your laboratory protocols in protocols.io to enhance the reproducibility of your results. Protocols.io assigns your protocol its own identifier (DOI) so that it can be cited independently in the future. For instructions see: https://journals.plos.org/plosone/s/submission-guidelines#loc-laboratory-protocols. Additionally, PLOS ONE offers an option for publishing peer-reviewed Lab Protocol articles, which describe protocols hosted on protocols.io. Read more information on sharing protocols at https://plos.org/protocols?utm_medium=editorial-email&utm_source=authorletters&utm_campaign=protocols.

As the corresponding author, your ORCID iD is verified in the submission system and will appear in the published article. PLOS supports the use of ORCID, and we encourage all coauthors to register for an ORCID iD and use it as well. Please encourage your coauthors to verify their ORCID iD within the submission system before final acceptance, as unverified ORCID iDs will not appear in the published article. *Only*  the individual author can complete the verification step; PLOS staff *cannot*  verify ORCID iDs on behalf of authors.

We look forward to receiving your revised manuscript.

Kind regards,

Udoka Okpalauwaekwe, MD, MPH, PhD

Academic Editor

PLOS One

Journal Requirements:

When submitting your revision, we need you to address these additional requirements.

1. Please ensure that your manuscript meets PLOS ONE's style requirements, including those for file naming. The PLOS ONE style templates can be found at

https://journals.plos.org/plosone/s/file?id=wjVg/PLOSOne_formatting_sample_main_body.pdf and

https://journals.plos.org/plosone/s/file?id=ba62/PLOSOne_formatting_sample_title_authors_affiliations.pdf

2. We note that you have indicated that there are restrictions to data sharing for this study. For studies involving human research participant data or other sensitive data, we encourage authors to share de-identified or anonymized data. However, when data cannot be publicly shared for ethical reasons, we allow authors to make their data sets available upon request. For information on unacceptable data access restrictions, please see http://journals.plos.org/plosone/s/data-availability#loc-unacceptable-data-access-restrictions.

Before we proceed with your manuscript, please address the following prompts:

a) If there are ethical or legal restrictions on sharing a de-identified data set, please explain them in detail (e.g., data contain potentially identifying or sensitive patient information, data are owned by a third-party organization, etc.) and who has imposed them (e.g., a Research Ethics Committee or Institutional Review Board, etc.). Please also provide contact information for a data access committee, ethics committee, or other institutional body to which data requests may be sent.

b) If there are no restrictions, please upload the minimal anonymized data set necessary to replicate your study findings to a stable, public repository and provide us with the relevant URLs, DOIs, or accession numbers. Please see http://www.bmj.com/content/340/bmj.c181.long for guidelines on how to de-identify and prepare clinical data for publication. For a list of recommended repositories, please see https://journals.plos.org/plosone/s/recommended-repositories. You also have the option of uploading the data as Supporting Information files, but we would recommend depositing data directly to a data repository if possible.

Please update your Data Availability statement in the submission form accordingly.

3. If the reviewer comments include a recommendation to cite specific previously published works, please review and evaluate these publications to determine whether they are relevant and should be cited. There is no requirement to cite these works unless the editor has indicated otherwise.

4. Please review your reference list to ensure that it is complete and correct. If you have cited papers that have been retracted, please include the rationale for doing so in the manuscript text, or remove these references and replace them with relevant current references. Any changes to the reference list should be mentioned in the rebuttal letter that accompanies your revised manuscript. If you need to cite a retracted article, indicate the article’s retracted status in the References list and also include a citation and full reference for the retraction notice.

Additional Editor Comments :

Well written protocol but conceptually over-engineered. The simultaneous use of multiple frameworks (14-component communication model, ISPIN-IBIN, and cultural transformation framework) risks adding complexity without clear justification of how each uniquely contributes to the analysis.

Relatedly, it is unclear whether these frameworks are guiding inclusion, analysis, or both, and how overlap between them will be managed.

The definition of “strategic communication” is broad and somewhat circular, which may introduce inconsistency in study selection.

Inclusion of opinion pieces and editorials raises concerns about conceptual dilution unless clearly distinguished from empirical evidence in synthesis.

While methodologically sound, your protocol leans heavily toward mapping rather than critical synthesis, which may limit the eventual contribution.

[Note: HTML markup is below. Please do not edit.]

Reviewers' comments:

Reviewer's Responses to Questions

**Comments to the Author**

1. Does the manuscript provide a valid rationale for the proposed study, with clearly identified and justified research questions?

Reviewer #1: Yes

**********

2. Is the protocol technically sound and planned in a manner that will lead to a meaningful outcome and allow testing the stated hypotheses?

Reviewer #1: Yes

**********

3. Is the methodology feasible and described in sufficient detail to allow the work to be replicable?

Reviewer #1: Yes

**********

4. Have the authors described where all data underlying the findings will be made available when the study is complete?

The PLOS Data policy

Reviewer #1: Yes

**********

5. Is the manuscript presented in an intelligible fashion and written in standard English?

Reviewer #1: Yes

**********

Please use the space provided to explain your answers to the questions above and, if applicable, provide comments about issues authors must address before this protocol can be accepted for publication. You may also include additional comments for the author, including concerns about research or publication ethics.

You may also provide optional suggestions and comments to authors that they might find helpful in planning their study.

Reviewer #1: Consolidated Analysis Report of the Scoping Review Protocol

I. General Observations

General Strengths:

• Methodological Rigor: Strong adherence to JBI methodology for scoping reviews and commitment to PRISMA-ScR reporting guidelines, ensuring transparency, reproducibility, and quality of the review.

• In-depth Conceptualization: The proposal to utilize three complementary analytical frameworks (a 14-component strategic communication framework, a 4-domain ISPIN-IBIN model for professional identity, and an 8-dimension cultural transformation framework) to guide study selection and data extraction is a major differentiating factor, enabling a multifaceted and rich analysis of the phenomenon under study.

• Transparency and Accountability: The registration of the protocol on the Open Science Framework (OSF) with a specific DOI enhances the transparency of the process and the accountability of the authors.

• Comprehensive Search: The search strategy will be conducted across seven bibliographic databases and complemented by grey literature searches, with no language restrictions, maximizing the retrieval of relevant literature.

General Weaknesses:

• Absence of Detailed Timeline: The lack of an explicit timeline for the review phases may hinder project planning and progress monitoring.

• Post-Synthesis Expert Consultation: While expert consultation was conducted during the initial phases, the absence of a formal post-synthesis stakeholder consultation step to validate preliminary findings and refine recommendations represents a missed opportunity to enrich the results and enhance their practical applicability.

General Suggestions for Improvement:

• Incorporate an Explicit Timeline: Adding a detailed timeline for all review phases would aid project management and communication of progress.

• Consider Expert Consultation for Validation: Evaluate the inclusion of a formal expert or stakeholder consultation step (such as the optional fifth stage of the JBI methodology for scoping reviews) to validate preliminary results and the practical implications of the review.

II. Analysis by Specific Manuscript Sections/Points

1. Presentation of Results

• Strengths:

o Adequate and Detailed Plan: The protocol presents a clear and multifaceted preliminary plan for the presentation of results, which is ADEQUATE. The authors specify that they will use:

� Tables and Figures: To summarize quantitative data (such as publication year, country, study design, setting), as indicated in PONE-D-25-53788_reviewer, Page 12: "Quantitative data (e.g., publication year, country, study design, setting) will be summarized in tables and figures to map the evidence."

� Conceptual Models or Evidence-Mapping Diagrams: To illustrate relationships between communication strategies, contextual factors, and outcomes. PONE-D-25-53788_reviewer, Page 12, states: "Conceptual models or evidence-mapping diagrams may be developed to illustrate relationships among communication strategies, contextual factors, and outcomes."

� Descriptive Tables and Narrative Summaries: To systematically map the results to the three analytical frameworks (the 14-component strategic communication model, the four-domain ISPIN-IBIN framework, and the eight-dimension cultural transformation framework). As per PONE-D-25-53788_reviewer, Page 13: "Results will be presented in descriptive tables and narrative summaries, systematically mapped to the 14-component strategic communication model, the four-domain ISPIN-IBIN framework, and the eight-dimension cultural transformation framework."

o Clarity and Structure: The manner in which results will be presented is well-articulated, directly linking to the operational frameworks that will guide the analysis.

• Weaknesses:

o No weaknesses were identified in this aspect, as the presentation plan is comprehensive and well-described.

• Suggestions for Improvement:

o No additional suggestions are needed for this point, as the current description is robust and well-planned. Consistent implementation of this plan will be crucial.

2. Expert Consultation

• Strengths:

o Robust Initial Consultation: The team demonstrated ADEQUACY by conducting expert consultations during crucial phases of protocol development, which is a strong point.

� The 14-component strategic communication framework was "refined through expert consultation and pilot testing" (PONE-D-25-53788_reviewer, Page 6).

� The search strategy was "developed with a university librarian and peer-reviewed via PRESS" (PONE-D-25-53788_reviewer, Page 11), and specific acknowledgment is given to Professor Richard Ricciardi for "expert insights and critical discussion of the topic, as well as his feedback on the preliminary search strategy" (PONE-D-25-53788_reviewer, Page 17).

o Dissemination Plan for Stakeholders: There is a plan for "stakeholder briefings for nursing leadership, organizational development, and health communication communities" (PONE-D-25-53788_reviewer, Page 16), ensuring that findings will reach the relevant audience.

• Weaknesses:

o Absence of Formal Post-Synthesis Consultation: The main weakness is the absence of a formal and explicit plan for an expert or stakeholder consultation step as an additional stage of the scoping review itself, specifically to validate or refine preliminary findings and recommendations. JBI methodology for scoping reviews often includes this optional stage (the fifth stage) to enhance the relevance and applicability of the findings. The consultation mentioned in the protocol occurred before the review (protocol development) or is planned for after the review's completion (dissemination).

• Suggestions for Improvement:

o Include a Formal Post-Synthesis Consultation Step: It is recommended to consider including a formal expert or stakeholder consultation step after the preliminary synthesis of results. This step could involve presenting the findings to a panel of experts (nurses, healthcare leaders, communication specialists) to gather feedback on data interpretation, identify additional gaps, and validate the relevance and practicality of the implications and recommendations. This would further align the protocol with best practices for scoping reviews and enhance the impact of the results.

3. Protocol Registration

• Strengths:

o Appropriate Registration: The protocol was ADEQUATELY registered, which is a point of research excellence.

o Transparency and Reproducibility: Registration on the Open Science Framework (OSF) with a specific DOI (Digital Object Identifier) strongly indicates a commitment to research transparency and reproducibility. As per PONE-D-25-53788_reviewer, Page 9: "The protocol is registered on the Open Science Framework, with any amendments documented publicly [38]." And in PONE-D-25-53788_reviewer, Page 16: "The protocol has been prospectively registered on the Open Science Framework (OSF): https://doi.org/10.17605/OSF.IO/VHC4K".

• Weaknesses:

o No weaknesses identified.

• Suggestions for Improvement:

o No suggestions are necessary, as this aspect is excellently addressed.

4. PRISMA-ScR Compliance Statement

• Strengths:

o Clear Commitment to Guidelines: The compliance statement is ADEQUATE and explicit.

o Reporting Quality and Transparency: The clear mention that the review will follow PRISMA-ScR (Preferred Reporting Items for Systematic reviews and Meta-Analyses extension for Scoping Reviews) guidelines is a strong point. This ensures that the final report will be comprehensive, transparent, and of high quality, facilitating its interpretation and application by other researchers and professionals. In PONE-D-25-53788_reviewer, Page 9: "This review will follow the Joanna Briggs Institute (JBI) methodology for scoping reviews [35,36] and report according to PRISMA-ScR [37]." And reinforced in PONE-D-25-53788_reviewer, Page 15: "Adherence to established methodological standards (JBI framework, PRISMA-ScR guidelines)."

• Weaknesses:

o No weaknesses identified.

• Suggestions for Improvement:

o No suggestions are necessary, as this aspect is well addressed.

5. Timeline

• Strengths:

o No specific strengths related to a timeline were identified, as the protocol does not include one.

• Weaknesses:

o Absence of Explicit Timeline: The primary weakness is that the protocol DOES NOT include an explicit or detailed tentative timeline for the completion of the scoping review. While it mentions the cutoff date for the literature to be searched (2000 to 2025), this refers to the period of literature inclusion, not the execution time of the review project itself. The lack of estimated phases (search, screening, extraction, analysis, writing, etc.) can hinder planning and time management.

• Suggestions for Improvement:

o Add a Detailed Timeline: It is essential to add a detailed tentative timeline specifying the estimated durations for each phase of the review. This not only aids in project planning and execution but also enhances the transparency of the protocol. Below, I provide a timeline template that could be adapted and included:

Tentative Scoping Review Timeline Template

This timeline is an estimate and may be adjusted based on the complexity of the literature, team availability, and unexpected challenges that may arise.

Stage Description Estimated Duration Suggested Period (Example) Responsible(s)

Phase 1: Preparation

1.1. Protocol Development & Search Strategy Refinement Defining objectives, review questions, eligibility criteria, and detailed search strategy. (Already completed, but important for formal time recording). 4 weeks Month 1 Carmen Arteaga-Jordá (CAJ), Hildegart González-Luis (HGL), Librarian

1.2. Protocol Registration Formal registration of the protocol on a platform like OSF. (Already completed). 1 week Month 1 CAJ, HGL

Phase 2: Search and Selection

2.1. Database Search Execution Executing comprehensive search strategies across seven bibliographic databases and grey literature. 2 weeks Month 2 CAJ

2.2. Reference Management & Deduplication Importing all search results into Covidence and removing duplicates. 1 week Month 2 CAJ

2.3. Title and Abstract Screening Independent screening of titles and abstracts by two reviewers (blinded). Conflict resolution. 4-6 weeks Month 3 - Month 4 CAJ, María Jesús Yáñez-Galdames (MJYG) (and HGL for conflicts)

2.4. Full-Text Screening Retrieval and independent full-text screening of eligible studies. Documentation of exclusion reasons. Conflict resolution. 4-6 weeks Month 4 - Month 5 CAJ, MJYG (and HGL for conflicts)

Phase 3: Extraction and Analysis

3.1. Data Extraction Tool Calibration Conducting final calibration and piloting of the data extraction tool (if necessary, beyond initial piloting). 1 week Month 6 CAJ, MJYG

3.2. Data Extraction Independent data extraction from included studies using the standardized tool. Contacting authors for missing information. 6-8 weeks Month 6 - Month 7 CAJ, María José Hernández-Leal (MJHL)

3.3. Data Analysis (Quantitative & Qualitative) Descriptive analysis of quantitative data and thematic analysis of qualitative data. Glossary development. 4-6 weeks Month 8 - Month 9 CAJ, HGL, Cristina Oroviogoicoechea Ortega (COO)

3.4. Convergent Synthesis Integration of quantitative and qualitative findings, mapping them to the frameworks. Development of conceptual models/diagrams. 3 weeks Month 9 - Month 10 CAJ, HGL, COO

Phase 4: Dissemination

4.1. Manuscript Drafting Drafting the initial manuscript of the scoping review, including results, discussion, and conclusion sections. 6 weeks Month 10 - Month 11 CAJ (initial draft), All authors (review & editing)

4.2. Internal Review and Feedback Critical review of the manuscript by co-authors and incorporation of feedback. 3 weeks Month 12 All authors

4.3. Preparation for Submission Formatting, reference checking, preparation of figures and tables, etc. 1 week Month 12 - Month 13 CAJ

4.4. Journal Submission & Post-Submission Revisions Submission of the manuscript to a peer-reviewed journal and response to reviewers. Variable (3-6 months or more) From Month 13 onwards All authors

4.5. Presentations and Stakeholder Briefings Preparation and delivery of conference presentations and briefings for stakeholders. Ongoing From Month 13 onwards All authors

6. Funding

• Strengths:

o Full Transparency: Funding declarations are ADEQUATE and provided clearly and explicitly in two sections of the protocol. In PONE-D-25-53788_reviewer, Page 2, under "Financial Disclosure," it states: "The author(s) received no specific funding for this work." And again, in PONE-D-25-53788_reviewer, Page 16, under the "Funding" section: "This scoping review did not receive any specific grant from funding agencies in the public, commercial, or not-for-profit sectors." This duplication reinforces the clarity of the information.

• Weaknesses:

o No weaknesses identified.

• Suggestions for Improvement:

o No suggestions are necessary, as this aspect is perfectly addressed.

7. Conflicts of Interest

• Strengths:

o Clear Declaration: The declaration of potential conflicts of interest is ADEQUATE and transparent. Similar to funding, the information is provided in two sections of the protocol, ensuring no ambiguity. In PONE-D-25-53788_reviewer, Page 2, under the "Competing Interests" section, the authors declare: "The authors have declared that no competing interests exist." And in PONE-D-25-53788_reviewer, Page 16, under the "Conflicts of interest" section: "The authors declare no competing interests."

• Weaknesses:

o No weaknesses identified.

• Suggestions for Improvement:

o No suggestions are necessary, as this aspect is very well addressed.

8. Appendices: Detailed Search Strategy

• Strengths:

o Adequate Planning: The protocol demonstrates ADEQUACY by planning to include the detailed search strategy as an appendix.

o Search Reproducibility: The clear mention that the "detailed search strategy for at least one major database" will be included in Appendix S5 is crucial for the review's reproducibility. In PONE-D-25-53788_reviewer, Page 10, the "Search strategy" section refers to "(S5 Appendix)," and PONE-D-25-53788_reviewer, Page 23, specifies: "S5 Appendix. Full search strategy. Complete database search strings, PRESS peer-review documentation, and OSF DOI for protocol registration."

• Weaknesses:

o No weaknesses identified.

• Suggestions for Improvement:

o No suggestions are necessary, as this aspect is perfectly addressed in the planning.

9. Appendices: Data Extraction Instrument

• Strengths:

o Adequate Planning: The protocol demonstrates ADEQUACY by planning to include the data extraction instrument as an appendix.

o Extraction Transparency: The inclusion of the data extraction instrument (Appendix S6) is a strong point that promotes transparency and reproducibility of the extraction process. As per PONE-D-25-53788_reviewer, Page 12, in "Data extraction," the authors state: "(see S6 Appendix)." And PONE-D-25-53788_reviewer, Page 23, in "Supporting information captions," specifies: "S6 Appendix. Integrated data extraction tool. Standardized, piloted extraction form adapted from JBI methodology for charting study data."

• Weaknesses:

o No weaknesses identified.

• Suggestions for Improvement:

o No suggestions are necessary, as this aspect is perfectly addressed in the planning.

**********

what does this mean?). If published, this will include your full peer review and any attached files.

If you choose “no”, your identity will remain anonymous but your review may still be made public.

**Do you want your identity to be public for this peer review?**  For information about this choice, including consent withdrawal, please see our Privacy Policy

Reviewer #1: **Yes:**  Emerson Roberto dos Santos, MsS.

**********

[NOTE: If reviewer comments were submitted as an attachment file, they will be attached to this email and accessible via the submission site. Please log into your account, locate the manuscript record, and check for the action link "View Attachments". If this link does not appear, there are no attachment files.]

To ensure your figures meet our technical requirements, please review our figure guidelines: https://journals.plos.org/plosone/s/figures

You may also use PLOS’s free figure tool, NAAS, to help you prepare publication quality figures: https://journals.plos.org/plosone/s/figures#loc-tools-for-figure-preparation.

NAAS will assess whether your figures meet our technical requirements by comparing each figure against our figure specifications.

Attachment Submitted filename: Consolidated Analysis Report of the Scoping Review Protocol.pdf

# Author comment: Author response to Decision Letter 1

**DOI:** 10.1371/journal.pone.0359546.r002

18 May 2026

RE: Resubmission of PONE-D-25-53788

Dear Dr. Okpalauwaekwe,

We are pleased to resubmit our manuscript entitled:

“The role of strategic communication in integrating cultural change and reinforcing nurses’ professional identity in healthcare organizations: a scoping review protocol” (PONE-D-25-53788) for consideration in PLOS ONE.

We sincerely thank you and Reviewer #1 (Emerson Roberto dos Santos, MSc) for the constructive and insightful feedback. The revisions have significantly enhanced the manuscript's conceptual clarity, methodological transparency, and analytical depth, while addressing all points raised.

In accordance with your recommendations, we have:

● Clarified the interaction between the three core concepts—strategic communication, cultural transformation, and nurses’ professional identity—and their distinct roles in inclusion criteria, data extraction, and analysis, with explicit management of framework overlaps.

● Refined the operational definition of strategic communication as intentional, organization-level practices aligned with institutional goals to influence professional identity and organizational culture, ensuring consistency during screening.

● Strengthened the analytical contribution by incorporating an explicit interpretative layer within the convergent synthesis, enabling the exploration of relationships beyond descriptive mapping.

● Clarified the handling of opinion pieces and editorials: these will be included only when providing substantive conceptual contributions, analysed separately from empirical studies, and clearly identified in the synthesis (none are currently expected based on preliminary screening).

● Incorporated an optional post-synthesis expert consultation step to enhance interpretative rigor and practical relevance.

● Added a tentative timeline for the review phases as Supplementary Information (S5 Appendix).

These revisions are minor in nature, do not alter the core study design or methodology, and are fully detailed in the accompanying point-by-point Response to Reviewers document and visible throughout the tracked-changes manuscript.

Submitted Files:

- Clean version (Manuscript)

- Tracked-changes version of the manuscript

- Point-by-point Response to Reviewers

Data Availability Statement: Data sharing is not applicable to this article as no datasets were generated or analyzed during the current scoping review protocol.

We confirm that this manuscript remains original, unpublished, and approved by all authors.

Thank you again for your time and continued consideration of our work. We look forward to your response.

Sincerely,

Hildegart González-Luis, PhD

On behalf of all co-authors

Dear Smiley Joyce Samson,

Thank you for your careful review of our submission and for identifying these issues.

We have revised the manuscript accordingly and addressed all points raised.

• Editorial Comment 1:

“Please amend your manuscript to include a reference list. References must be placed at the end of the manuscript and numbered in the order that they appear in the text.”

Response:

We revised the manuscript to ensure full compliance with PLOS ONE reference formatting requirements. The supplementary reference document (S8 Appendix: References for Supporting Information), which previously included only references not cited in the main text, has now been expanded to provide a complete and independent reference list for all supplementary appendices, ensuring that the supplementary material is fully self-contained. All supplementary references have also been standardized to Vancouver style.

• Editorial Comment 2:

“Please ensure that you refer to Table 2 in your text as, if accepted, production will need this reference to link the reader to the Table.”

Response:

We thank the Editorial Team for identifying this inconsistency. During the revision process, a table numbering error was inadvertently introduced. The manuscript contains only two tables in the main text, while the remaining tables were transferred to the supplementary appendices. Following correction, the previous “Table 3” is now correctly labelled as “Table 2,” and all corresponding in-text citations have been updated accordingly (see clean manuscript, lines 237 and 242).

• Editorial Comment 3:

“Please include a copy of Table 2 which you refer to in your text.”

Response:

The issue resulted from the same table numbering inconsistency described above. No new table has been introduced. The previous “Table 3” has now been correctly updated and incorporated as “Table 2” in the revised manuscript.

Thank you again for your time and consideration.

Kind regards,

Hildegart González-Luis, PhD

On behalf of all co-authors

Attachment Submitted filename: Response to Review Decision.docx

# Peer review: Decision Letter 1

**DOI:** 10.1371/journal.pone.0359546.r003

**Authors:** Udoka Okpalauwaekwe

12 Jun 2026

Dear Dr. González Luis,

Thank you for submitting your manuscript to PLOS ONE. After careful consideration, we feel that it has merit but does not fully meet PLOS ONE’s publication criteria as it currently stands. Therefore, we invite you to submit a revised version of the manuscript that addresses the points raised during the review process.

Please submit your revised manuscript by Jul 27 2026 11:59PM. If you will need more time than this to complete your revisions, please reply to this message or contact the journal office at plosone@plos.org. When you're ready to submit your revision, log on to https://www.editorialmanager.com/pone/ and select the 'Submissions Needing Revision' folder to locate your manuscript file.

- A letter that responds to each point raised by the academic editor and reviewer(s). You should upload this letter as a separate file labeled 'Response to Reviewers'.
- A marked-up copy of your manuscript that highlights changes made to the original version. You should upload this as a separate file labeled 'Revised Manuscript with Track Changes'.
- An unmarked version of your revised paper without tracked changes. You should upload this as a separate file labeled 'Manuscript'.

If you would like to make changes to your financial disclosure, please include your updated statement in your cover letter. Guidelines for resubmitting your figure files are available below the reviewer comments at the end of this letter.

If applicable, we recommend that you deposit your laboratory protocols in protocols.io to enhance the reproducibility of your results. Protocols.io assigns your protocol its own identifier (DOI) so that it can be cited independently in the future. For instructions see: https://journals.plos.org/plosone/s/submission-guidelines#loc-laboratory-protocols. Additionally, PLOS ONE offers an option for publishing peer-reviewed Lab Protocol articles, which describe protocols hosted on protocols.io. Read more information on sharing protocols at https://plos.org/protocols?utm_medium=editorial-email&utm_source=authorletters&utm_campaign=protocols.

As the corresponding author, your ORCID iD is verified in the submission system and will appear in the published article. PLOS supports the use of ORCID, and we encourage all coauthors to register for an ORCID iD and use it as well. Please encourage your coauthors to verify their ORCID iD within the submission system before final acceptance, as unverified ORCID iDs will not appear in the published article. *Only*  the individual author can complete the verification step; PLOS staff *cannot*  verify ORCID iDs on behalf of authors.

We look forward to receiving your revised manuscript.

Kind regards,

Udoka Okpalauwaekwe, MD, MPH, PhD

Academic Editor

PLOS One

Journal Requirements:

If the reviewer comments include a recommendation to cite specific previously published works, please review and evaluate these publications to determine whether they are relevant and should be cited. There is no requirement to cite these works unless the editor has indicated otherwise.

Please review your reference list to ensure that it is complete and correct. If you have cited papers that have been retracted, please include the rationale for doing so in the manuscript text, or remove these references and replace them with relevant current references. Any changes to the reference list should be mentioned in the rebuttal letter that accompanies your revised manuscript. If you need to cite a retracted article, indicate the article’s retracted status in the References list and also include a citation and full reference for the retraction notice.

[Note: HTML markup is below. Please do not edit.]

Reviewers' comments:

Reviewer's Responses to Questions

**Comments to the Author**

1. Does the manuscript provide a valid rationale for the proposed study, with clearly identified and justified research questions?

Reviewer #1: Yes

**********

2. Is the protocol technically sound and planned in a manner that will lead to a meaningful outcome and allow testing the stated hypotheses?

Reviewer #1: Partly

**********

3. Is the methodology feasible and described in sufficient detail to allow the work to be replicable?

Reviewer #1: No

**********

4. Have the authors described where all data underlying the findings will be made available when the study is complete?

The PLOS Data policy

Reviewer #1: Yes

**********

5. Is the manuscript presented in an intelligible fashion and written in standard English?

Reviewer #1: Yes

**********

Please use the space provided to explain your answers to the questions above and, if applicable, provide comments about issues authors must address before this protocol can be accepted for publication. You may also include additional comments for the author, including concerns about research or publication ethics.

You may also provide optional suggestions and comments to authors that they might find helpful in planning their study.

Reviewer #1: FINAL ASSESSMENT

The revised manuscript demonstrates good progress in addressing the recommendations, with 55.6% full compliance and 88.9% overall compliance (including partially compliant items).

Strengths: Conceptual clarity, transparency, and compliance with international guidelines.

Critical areas requiring additional revision:

1. Detailed project timeline (non-compliant)

2. Specific expert consultation protocol (partially compliant)

3. Technical appendices (search strategy and data extraction instrument – partially compliant)

Recommendation: Request that authors prioritize addressing the non-compliant recommendation (Timeline) and detail the three partially compliant recommendations before final acceptance.

**********

what does this mean?). If published, this will include your full peer review and any attached files.

If you choose “no”, your identity will remain anonymous but your review may still be made public.

**Do you want your identity to be public for this peer review?**  For information about this choice, including consent withdrawal, please see our Privacy Policy

Reviewer #1: **Yes:**  Dos Santos, Emerson Roberto

**********

[NOTE: If reviewer comments were submitted as an attachment file, they will be attached to this email and accessible via the submission site. Please log into your account, locate the manuscript record, and check for the action link "View Attachments". If this link does not appear, there are no attachment files.]

To ensure your figures meet our technical requirements, please review our figure guidelines: https://journals.plos.org/plosone/s/figures

You may also use PLOS’s free figure tool, NAAS, to help you prepare publication quality figures: https://journals.plos.org/plosone/s/figures#loc-tools-for-figure-preparation.

NAAS will assess whether your figures meet our technical requirements by comparing each figure against our figure specifications.

Attachment Submitted filename: EVALUATION REPORT.pdf

# Author comment: Author response to Decision Letter 2

**DOI:** 10.1371/journal.pone.0359546.r004

17 Jun 2026

Hildegart González-Luis, PhD

University of Navarra

17th of June 2026

Udoka Okpalauwaekwe, MD, MPH, PhD

Academic Editor

PLOS One

RE: Resubmission of PONE-D-25-53788

Dear Dr. Okpalauwaekwe,

We are pleased to resubmit our manuscript entitled:

“The role of strategic communication in integrating cultural change and reinforcing nurses’ professional identity in healthcare organizations: a scoping review protocol” (PONE-D-25-53788) for consideration in PLOS ONE.

We sincerely thank you and Reviewer #1 (Emerson Roberto dos Santos, MsS) for the constructive and insightful feedback. The revisions have significantly enhanced the manuscript’s conceptual clarity, methodological transparency, and analytical rigor.

Following the reviewer’s final assessment, we have addressed all remaining points identified as non-compliant or partially compliant. Specifically, we have:

• Expanded the description of the expert consultation phase, including its timing, expert selection criteria, feedback collection approach, and intended contribution to interpretation of findings.

• Clarified the availability and location of the detailed review timeline provided in the Supplementary Information (S5 Appendix).

• Enhanced transparency of the search process by adding a database-specific search adaptation framework (S7 Appendix) describing the implementation of the validated search strategy across all information sources.

• Further detailed the data extraction process by clarifying the content of the data extraction instrument and coding guide (S8 Appendix) and including an illustrative example of application.

These revisions do not alter the core study design or methodology but provide additional detail and transparency in response to the reviewer’s recommendations. All modifications are documented in the accompanying Response to Reviewer Comments document and are visible in the tracked-changes version of the manuscript.

Submitted Files:

• Manuscript (clean version)

• Revised Manuscript with Track Changes

• Suplementary Information

• Suplementary Information with Track Changes

• Response to Reviewer Comments

We confirm that this manuscript remains original, unpublished, and approved by all authors.

Thank you again for your time and consideration. We look forward to your response.

Sincerely,

Hildegart González-Luis, PhD

On behalf of all co-authors

Attachment Submitted filename: Response to Reviewers .docx

# Peer review: Decision Letter 2

**DOI:** 10.1371/journal.pone.0359546.r005

**Authors:** Udoka Okpalauwaekwe

1 Jul 2026

Dear Dr. González Luis,

Thank you for submitting your manuscript to PLOS ONE. After careful consideration, we feel that it has merit but does not fully meet PLOS ONE’s publication criteria as it currently stands. Therefore, we invite you to submit a revised version of the manuscript that addresses the points raised during the review process.

Please submit your revised manuscript by Aug 15 2026 11:59PM. If you will need more time than this to complete your revisions, please reply to this message or contact the journal office at plosone@plos.org. When you're ready to submit your revision, log on to https://www.editorialmanager.com/pone/ and select the 'Submissions Needing Revision' folder to locate your manuscript file.

- A letter that responds to each point raised by the academic editor and reviewer(s). You should upload this letter as a separate file labeled 'Response to Reviewers'.
- A marked-up copy of your manuscript that highlights changes made to the original version. You should upload this as a separate file labeled 'Revised Manuscript with Track Changes'.
- An unmarked version of your revised paper without tracked changes. You should upload this as a separate file labeled 'Manuscript'.

If you would like to make changes to your financial disclosure, please include your updated statement in your cover letter. Guidelines for resubmitting your figure files are available below the reviewer comments at the end of this letter.

If applicable, we recommend that you deposit your laboratory protocols in protocols.io to enhance the reproducibility of your results. Protocols.io assigns your protocol its own identifier (DOI) so that it can be cited independently in the future. For instructions see: https://journals.plos.org/plosone/s/submission-guidelines#loc-laboratory-protocols. Additionally, PLOS ONE offers an option for publishing peer-reviewed Lab Protocol articles, which describe protocols hosted on protocols.io. Read more information on sharing protocols at https://plos.org/protocols?utm_medium=editorial-email&utm_source=authorletters&utm_campaign=protocols.

As the corresponding author, your ORCID iD is verified in the submission system and will appear in the published article. PLOS supports the use of ORCID, and we encourage all coauthors to register for an ORCID iD and use it as well. Please encourage your coauthors to verify their ORCID iD within the submission system before final acceptance, as unverified ORCID iDs will not appear in the published article. *Only*  the individual author can complete the verification step; PLOS staff *cannot*  verify ORCID iDs on behalf of authors.

We look forward to receiving your revised manuscript.

Kind regards,

Udoka Okpalauwaekwe, MD, MPH, PhD

Academic Editor

PLOS One

Journal Requirements:

If the reviewer comments include a recommendation to cite specific previously published works, please review and evaluate these publications to determine whether they are relevant and should be cited. There is no requirement to cite these works unless the editor has indicated otherwise.

Please review your reference list to ensure that it is complete and correct. If you have cited papers that have been retracted, please include the rationale for doing so in the manuscript text, or remove these references and replace them with relevant current references. Any changes to the reference list should be mentioned in the rebuttal letter that accompanies your revised manuscript. If you need to cite a retracted article, indicate the article’s retracted status in the References list and also include a citation and full reference for the retraction notice.

[Note: HTML markup is below. Please do not edit.]

Reviewers' comments:

Reviewer's Responses to Questions

**Comments to the Author**

1. Does the manuscript provide a valid rationale for the proposed study, with clearly identified and justified research questions?

Reviewer #1: Yes

**********

2. Is the protocol technically sound and planned in a manner that will lead to a meaningful outcome and allow testing the stated hypotheses?

Reviewer #1: Yes

**********

3. Is the methodology feasible and described in sufficient detail to allow the work to be replicable?

Reviewer #1: Yes

**********

4. Have the authors described where all data underlying the findings will be made available when the study is complete?

The PLOS Data policy

Reviewer #1: Yes

**********

5. Is the manuscript presented in an intelligible fashion and written in standard English?

Reviewer #1: Yes

**********

Please use the space provided to explain your answers to the questions above and, if applicable, provide comments about issues authors must address before this protocol can be accepted for publication. You may also include additional comments for the author, including concerns about research or publication ethics.

You may also provide optional suggestions and comments to authors that they might find helpful in planning their study.

Reviewer #1: This manuscript presents a methodologically sound scoping review protocol, following the Joanna Briggs Institute (JBI) methodology and the PRISMA-ScR guidelines. The review proposes to map the literature on how strategic communication can strengthen nurses' professional identity during processes of cultural transformation in healthcare organizations. The majority of items assessed were classified as Adequate, with a few items identified as Requires revision . The protocol is consistent, well-structured, and demonstrates methodological maturity, representing a revised submission (R2) that has already incorporated feedback from prior rounds of peer review.

**********

what does this mean?). If published, this will include your full peer review and any attached files.

If you choose “no”, your identity will remain anonymous but your review may still be made public.

**Do you want your identity to be public for this peer review?**  For information about this choice, including consent withdrawal, please see our Privacy Policy

Reviewer #1: **Yes:**  EMERSON ROBERTO DOS SANTOS

**********

[NOTE: If reviewer comments were submitted as an attachment file, they will be attached to this email and accessible via the submission site. Please log into your account, locate the manuscript record, and check for the action link "View Attachments". If this link does not appear, there are no attachment files.]

To ensure your figures meet our technical requirements, please review our figure guidelines: https://journals.plos.org/plosone/s/figures

You may also use PLOS’s free figure tool, NAAS, to help you prepare publication quality figures: https://journals.plos.org/plosone/s/figures#loc-tools-for-figure-preparation.

NAAS will assess whether your figures meet our technical requirements by comparing each figure against our figure specifications.

Attachment Submitted filename: renamed_e82c1.pdf

# Author comment: Author response to Decision Letter 3

**DOI:** 10.1371/journal.pone.0359546.r006

14 Jul 2026

Hildegart González-Luis, PhD

University of Navarra

14th of July 2026

Udoka Okpalauwaekwe, MD, MPH, PhD

Academic Editor

PLOS One

RE: Resubmission of PONE-D-25-53788

Dear Dr. Okpalauwaekwe,

We are pleased to resubmit our manuscript entitled “The role of strategic communication in integrating cultural change and reinforcing nurses' professional identity in healthcare organizations: a scoping review protocol” (PONE-D-25-53788) for consideration in PLOS ONE.

We thank you for your continued consideration of our manuscript and for the reviewer’s final feedback. In response to the comments and recommendations received, we have revised the manuscript to improve transparency, reproducibility, and ease of verification, without altering the study design or methodology.

Specifically, the revised submission:

• Clarifies in the manuscript the location and consistency of the Supplementary Information, including the complete search strategy, database-specific search adaptations, and data extraction instrument;

• Expands the Strengths and Limitations section to address publication bias, language bias, conceptual heterogeneity, the absence of formal critical appraisal, and the interpretative nature of narrative synthesis, together with the methodological safeguards adopted.

A point-by-point Response to Reviewer Comments is submitted as a separate file, and all corresponding manuscript changes are highlighted in the tracked-changes version.

Submitted Files

• Manuscript (clean version)

• Revised Manuscript with Track Changes

• Supplementary Information

• Response to Reviewer Comments

We confirm that the manuscript is original, unpublished, and approved by all authors.

Thank you again for your time and consideration.

Sincerely,

Hildegart González-Luis, PhD

On behalf of all co-authors

Attachment Submitted filename: Response to Reviewers.docx

# Peer review: Decision Letter 3

**DOI:** 10.1371/journal.pone.0359546.r007

**Authors:** Udoka Okpalauwaekwe

6 Aug 2026

Dear Dr. González Luis,

Thank you for submitting your manuscript to PLOS ONE. After careful consideration, we feel that it has merit but does not fully meet PLOS ONE’s publication criteria as it currently stands. Therefore, we invite you to submit a revised version of the manuscript that addresses the points raised during the review process.

Please submit your revised manuscript by Sep 20 2026 11:59PM. If you will need more time than this to complete your revisions, please reply to this message or contact the journal office at plosone@plos.org. When you're ready to submit your revision, log on to https://www.editorialmanager.com/pone/ and select the 'Submissions Needing Revision' folder to locate your manuscript file.

- A letter that responds to each point raised by the academic editor and reviewer(s). You should upload this letter as a separate file labeled 'Response to Reviewers'.
- A marked-up copy of your manuscript that highlights changes made to the original version. You should upload this as a separate file labeled 'Revised Manuscript with Track Changes'.
- An unmarked version of your revised paper without tracked changes. You should upload this as a separate file labeled 'Manuscript'.

If you would like to make changes to your financial disclosure, please include your updated statement in your cover letter. Guidelines for resubmitting your figure files are available below the reviewer comments at the end of this letter.

If applicable, we recommend that you deposit your laboratory protocols in protocols.io to enhance the reproducibility of your results. Protocols.io assigns your protocol its own identifier (DOI) so that it can be cited independently in the future. For instructions see: https://journals.plos.org/plosone/s/submission-guidelines#loc-laboratory-protocols. Additionally, PLOS ONE offers an option for publishing peer-reviewed Lab Protocol articles, which describe protocols hosted on protocols.io. Read more information on sharing protocols at https://plos.org/protocols?utm_medium=editorial-email&utm_source=authorletters&utm_campaign=protocols.

As the corresponding author, your ORCID iD is verified in the submission system and will appear in the published article. PLOS supports the use of ORCID, and we encourage all coauthors to register for an ORCID iD and use it as well. Please encourage your coauthors to verify their ORCID iD within the submission system before final acceptance, as unverified ORCID iDs will not appear in the published article. *Only*  the individual author can complete the verification step; PLOS staff *cannot*  verify ORCID iDs on behalf of authors.

We look forward to receiving your revised manuscript.

Kind regards,

Udoka Okpalauwaekwe, MD, MPH, PhD

Academic Editor

PLOS One

Journal Requirements:

If the reviewer comments include a recommendation to cite specific previously published works, please review and evaluate these publications to determine whether they are relevant and should be cited. There is no requirement to cite these works unless the editor has indicated otherwise.

Please review your reference list to ensure that it is complete and correct. If you have cited papers that have been retracted, please include the rationale for doing so in the manuscript text, or remove these references and replace them with relevant current references. Any changes to the reference list should be mentioned in the rebuttal letter that accompanies your revised manuscript. If you need to cite a retracted article, indicate the article’s retracted status in the References list and also include a citation and full reference for the retraction notice.

[Note: HTML markup is below. Please do not edit.]

Reviewers' comments:

Reviewer's Responses to Questions

**Comments to the Author**

1. Does the manuscript provide a valid rationale for the proposed study, with clearly identified and justified research questions?

Reviewer #1: Yes

**********

2. Is the protocol technically sound and planned in a manner that will lead to a meaningful outcome and allow testing the stated hypotheses?

Reviewer #1: Yes

**********

3. Is the methodology feasible and described in sufficient detail to allow the work to be replicable?

Reviewer #1: Yes

**********

4. Have the authors described where all data underlying the findings will be made available when the study is complete?

The PLOS Data policy

Reviewer #1: Yes

**********

5. Is the manuscript presented in an intelligible fashion and written in standard English?

Reviewer #1: Yes

**********

Please use the space provided to explain your answers to the questions above and, if applicable, provide comments about issues authors must address before this protocol can be accepted for publication. You may also include additional comments for the author, including concerns about research or publication ethics.

You may also provide optional suggestions and comments to authors that they might find helpful in planning their study.

Reviewer #1: See Assessment of Author Revisions — Manuscript PONE-D-25-53788 (R2 → R3).

Recommendation Status

1. Full search strategy as appendix ✅ FULLY ADDRESSED

2. Data extraction instrument as appendix ✅ FULLY ADDRESSED

3. Discussion of limitations and biases ✅ FULLY ADDRESSED

4. Verification of all appendices in submission package ✅ FULLY ADDRESSED

5. Detailed expert consultation plan ❌ NOT ADDRESSED

**********

what does this mean?). If published, this will include your full peer review and any attached files.

If you choose “no”, your identity will remain anonymous but your review may still be made public.

**Do you want your identity to be public for this peer review?**  For information about this choice, including consent withdrawal, please see our Privacy Policy

Reviewer #1: **Yes:**  EMERSON ROBERTO DOS SANTOS

**********

[NOTE: If reviewer comments were submitted as an attachment file, they will be attached to this email and accessible via the submission site. Please log into your account, locate the manuscript record, and check for the action link "View Attachments". If this link does not appear, there are no attachment files.]

To ensure your figures meet our technical requirements, please review our figure guidelines: https://journals.plos.org/plosone/s/figures

You may also use PLOS’s free figure tool, NAAS, to help you prepare publication quality figures: https://journals.plos.org/plosone/s/figures#loc-tools-for-figure-preparation.

NAAS will assess whether your figures meet our technical requirements by comparing each figure against our figure specifications.

Attachment Submitted filename: renamed_7a7ea.pdf

# Author comment: Author response to Decision Letter 4

**DOI:** 10.1371/journal.pone.0359546.r008

26 Aug 2026

Reviewer comment: Expert consultation plan

The authors' response: We appreciate this constructive suggestion. In response to the reviewer's recommendation, we have further elaborated the expert consultation stage. The revised manuscript now specifies that one independent international expert will be invited, outlines the expert selection criteria, and describes the consultation method (written comments and/or a virtual consultation). These additions provide greater clarity regarding the consultation process while preserving its intended role as an interpretative step to enhance analytical rigor rather than to modify the review findings.

Location: Data Analysis and Presentation section, page 15, lines 274–287

Attachment Submitted filename: Response_to_Reviewers_auresp_4.docx

# Peer review: Decision Letter 4

**DOI:** 10.1371/journal.pone.0359546.r009

**Authors:** Udoka Okpalauwaekwe

16 Sep 2026

Strategic communication to strengthen nurses’ professional identity and drive cultural change in healthcare organizations: A JBI Scoping Review Protocol

PONE-D-25-53788R4

Dear Dr. González Luis,

We’re pleased to inform you that your manuscript has been judged scientifically suitable for publication and will be formally accepted for publication once it meets all outstanding technical requirements.

Within one week, you’ll receive an e-mail detailing the required amendments. When these have been addressed, you’ll receive a formal acceptance letter and your manuscript will be scheduled for publication.

An invoice will be generated when your article is formally accepted. Please note, if your institution has a publishing partnership with PLOS and your article meets the relevant criteria, all or part of your publication costs will be covered. Please make sure your user information is up-to-date by logging into Editorial Manager at Editorial Manager® and clicking the ‘Update My Information' link at the top of the page. For questions related to billing, please contact billing support.

If your institution or institutions have a press office, please notify them about your upcoming paper to help maximize its impact. If they’ll be preparing press materials, please inform our press team as soon as possible -- no later than 48 hours after receiving the formal acceptance. Your manuscript will remain under strict press embargo until 2 pm Eastern Time on the date of publication. For more information, please contact onepress@plos.org.

Kind regards,

Udoka Okpalauwaekwe, MD, MPH, PhD

Academic Editor

PLOS One

Additional Editor Comments (optional):

Reviewers' comments:

Reviewer's Responses to Questions

**Comments to the Author**

1. Does the manuscript provide a valid rationale for the proposed study, with clearly identified and justified research questions?

Reviewer #1: Yes

**********

2. Is the protocol technically sound and planned in a manner that will lead to a meaningful outcome and allow testing the stated hypotheses?

Reviewer #1: Yes

**********

3. Is the methodology feasible and described in sufficient detail to allow the work to be replicable?

Reviewer #1: Yes

**********

4. Have the authors described where all data underlying the findings will be made available when the study is complete?

The PLOS Data policy

Reviewer #1: Yes

**********

5. Is the manuscript presented in an intelligible fashion and written in standard English?

Reviewer #1: Yes

**********

Please use the space provided to explain your answers to the questions above and, if applicable, provide comments about issues authors must address before this protocol can be accepted for publication. You may also include additional comments for the author, including concerns about research or publication ethics.

You may also provide optional suggestions and comments to authors that they might find helpful in planning their study.

Reviewer #1: Assessment of Author Revisions — Manuscript PONE-D-25-53788 (R3 → R4)

This report evaluates each recommendation issued in our previous peer review of the scoping review protocol titled "Strategic communication to strengthen nurses' professional identity and drive cultural change in healthcare organizations: A JBI Scoping Review Protocol" (PONE-D-25-53788), against the revised version submitted as PONE-D-25-53788_R4. Each recommendation is classified as ADDRESSED, PARTIALLY ADDRESSED, or NOT ADDRESSED. For items marked as partially addressed, the specific remaining shortcomings are indicated.

________________________________________

Recommendation 1 — Full search strategy as appendix

What we requested: Include the complete search string for MEDLINE/PubMed (or another primary database) with MeSH terms, Boolean operators, free-text keywords, applied filters, and the number of results retrieved. The corresponding appendix should be verified and, if absent, added.

Status: ✅ ADDRESSED

The complete PubMed search strategy remains included in the submission package as supplementary material (S6 — Complete search strategy for PubMed), retained from the previous revision cycle. The requirement for transparency and reproducibility of the search process is satisfied.

________________________________________

Recommendation 2 — Data extraction instrument as appendix

What we requested: Make the complete data extraction form/template available as supplementary material (S5 Appendix), containing all fields to be extracted from each included study.

Status: ✅ ADDRESSED

The data extraction instrument remains available as S5 — Data extraction instrument. Methodological transparency in the data extraction stage is fulfilled.

________________________________________

Recommendation 3 — Discussion of limitations and biases

What we requested: Add a section or paragraph acknowledging the inherent limitations of the scoping review methodology — absence of critical quality appraisal, publication bias, language bias, and subjectivity of narrative synthesis — together with the mitigations adopted.

Status: ✅ ADDRESSED

The manuscript retains a dedicated Strengths and Limitations section that explicitly addresses the absence of critical quality appraisal, potential publication bias (overrepresentation of contexts with greater academic output, e.g. USA, Europe, Australia), and language bias, as well as the mitigations adopted: two independent reviewers, multiple databases, grey literature searching, and expert consultation.

________________________________________

Recommendation 4 — Verification of all cited appendices in the submission package

What we requested: Ensure that all appendices cited in the manuscript (S1–S5) are actually present in the submission package.

Status: ✅ ADDRESSED

The supplementary material was retained and further expanded — the supporting information captions now extend through S9 — with no apparent gaps in the package.

________________________________________

Recommendation 5 — Detailed expert consultation plan

What we requested: Specify the estimated number of experts to be consulted, the selection criteria, and the consultation method (e.g., Delphi technique, interviews, focus group). This was the only outstanding item from the previous review round.

Status: ✅ ADDRESSED

The R4 version now contains (i) a dedicated entry in the Response to Reviewers addressing the "Expert consultation plan" comment, with the authors' response, and (ii) a cross-reference to the detailed text in the Data Analysis and Presentation section (page 15, lines 274–287). A targeted revision was therefore made at precisely the point previously flagged.

Verification note: We recommend a literal check of page 15 (lines 274–287) to confirm that all three elements are explicitly stated — estimated number of experts, selection criteria, and consultation method. The structure confirms the existence and location of the revision; the literal verification of these three items is advisable before the final decision is issued.

________________________________________

Additional item — Management of bias (Section 1.4 of the previous report)

Status: ✅ ADDRESSED

This item remains absorbed by the newly added Strengths and Limitations section, which covers publication bias, language bias, the absence of critical quality appraisal, and the mitigations adopted.

________________________________________

Compliance Rate

Recommendation Status

1. Full search strategy as appendix ✅ ADDRESSED

2. Data extraction instrument as appendix ✅ ADDRESSED

3. Discussion of limitations and biases ✅ ADDRESSED

4. Verification of all appendices in submission package ✅ ADDRESSED

5. Detailed expert consultation plan ✅ ADDRESSED

Additional item — Management of bias ✅ ADDRESSED

• Recommendations assessed: 5

• ✅ Addressed: 5

• ⚠️ Partially addressed: 0

• ❌ Not addressed: 0

• Compliance rate: 100% (5 of 5) — including the additional management-of-bias item, 6 of 6.

________________________________________

Input for the Final Decision

The sole outstanding recommendation from the previous round (the expert consultation plan) has been addressed in this version, raising compliance from 80% (R3) to 100% (R4).

Two further elements reinforce editorial conformity in R4: the presence of an AI-tool usage disclosure in the Acknowledgements, and the expansion of the supplementary material.

With all recommendations addressed, the manuscript can proceed toward acceptance, at most conditioned on the literal verification of the three elements in Recommendation 5.

**********

what does this mean?). If published, this will include your full peer review and any attached files.

If you choose “no”, your identity will remain anonymous but your review may still be made public.

**Do you want your identity to be public for this peer review?**  For information about this choice, including consent withdrawal, please see our Privacy Policy

Reviewer #1: **Yes:**  EMERSON ROBERTO DOS SANTOS

**********

Attachment Submitted filename: renamed_38f16.pdf

# Editor report: Acceptance letter

**DOI:** 10.1371/journal.pone.0359546.r010

**Authors:** Udoka Okpalauwaekwe

PONE-D-25-53788R4

PLOS One

Dear Dr. González-Luis,

I'm pleased to inform you that your manuscript has been deemed suitable for publication in PLOS One. Congratulations! Your manuscript is now being handed over to our production team.

At this stage, our production department will prepare your paper for publication. This includes ensuring the following:

* All references, tables, and figures are properly cited

* All relevant supporting information is included in the manuscript submission,

* There are no issues that prevent the paper from being properly typeset

You will receive further instructions from the production team, including instructions on how to review your proof when it is ready. Please keep in mind that we are working through a large volume of accepted articles, so please give us a few days to review your paper and let you know the next and final steps.

Lastly, if your institution or institutions have a press office, please let them know about your upcoming paper now to help maximize its impact. If they'll be preparing press materials, please inform our press team within the next 48 hours. Your manuscript will remain under strict press embargo until 2 pm Eastern Time on the date of publication. For more information, please contact onepress@plos.org.

You will receive an invoice from PLOS for your publication fee after your manuscript has reached the completed accept phase. If you receive an email requesting payment before acceptance or for any other service, this may be a phishing scheme. Learn how to identify phishing emails and protect your accounts at https://explore.plos.org/phishing.

If we can help with anything else, please email us at customercare@plos.org.

Thank you for submitting your work to PLOS One and supporting open access.

Kind regards,

PLOS One Editorial Office Staff

on behalf of

Dr. Udoka Okpalauwaekwe

Academic Editor

PLOS One
